# Supplementary material for: A general framework for predicting the transcriptomic consequences of non-coding variation and small molecules
Source: PLoS Comput Biol. 2022 Apr 14;18(4):e1010028. doi: 10.1371/journal.pcbi.1010028 (PMC9041867; doi:10.1371/journal.pcbi.1010028)
Supplement: S1 Table — Generally, across most tissues and chromosomes, the larger the impact a position has on the mean abundance of the gene (as indicated by a higher peaBrain impact metric), the more evolutionary conserved it is (i.e. a positive coefficient). The notable exception is the nucleus accumbens (basal ganglia), where the opposite trend is noted (negative coefficients; in bold). All coefficients are significant (p < 10−16). The results were also consistent with the rank-normalized phyloP and peaBrain scores. Abbreviations: L, lower; U, upper. (DOCX) [file pcbi.1010028.s001.docx]

**Table S1. Tabulated summary of coefficients of the linear function modelling phyloP conservation scores as a function of tissue-specific peaBrain noncoding impact metric.** Generally, across most tissues and chromosomes, the larger the impact a position has on the mean abundance of the gene (as indicated by a higher peaBrain impact metric), the more evolutionary conserved it is (i.e. a positive coefficient). The notable exception is the nucleus accumbens (basal ganglia), where the opposite trend is noted (negative coefficients; in bold). All coefficients are significant (p < 10^-16^). The results were also consistent with the rank-normalized phyloP and peaBrain scores. **Abbreviations:** L, lower; U, upper.

|  | **Linear Model**  **Coefficient** | **L Bound** | **U Bound** |
| --- | --- | --- | --- |
| AdiposeSubcutaneous | 15.79 | 15.72 | 15.86 |
| AdiposeVisceralOmentum | 14.32 | 14.25 | 14.39 |
| AdrenalGland | 0.33 | 0.27 | 0.39 |
| ArteryAorta | 3.51 | 3.47 | 3.56 |
| ArteryCoronary | 5.64 | 5.59 | 5.69 |
| ArteryTibial | 5.89 | 5.84 | 5.94 |
| BrainAmygdala | 9.25 | 9.19 | 9.31 |
| BrainAnteriorcingulatecortexBA24 | 6.92 | 6.85 | 7.00 |
| BrainCaudatebasalganglia | 6.19 | 6.15 | 6.23 |
| BrainCerebellarHemisphere | 13.48 | 13.41 | 13.55 |
| BrainCerebellum | 4.78 | 4.73 | 4.83 |
| BrainCortex | 2.70 | 2.67 | 2.74 |
| BrainFrontalCortexBA9 | 8.57 | 8.50 | 8.64 |
| BrainHippocampus | 5.09 | 5.03 | 5.14 |
| BrainHypothalamus | 5.17 | 5.09 | 5.24 |
| **BrainNucleusaccumbensbasalganglia** | **-1.32** | **-1.37** | **-1.27** |
| BrainPutamenbasalganglia | 6.91 | 6.86 | 6.96 |
| BreastMammaryTissue | 4.24 | 4.19 | 4.30 |
| CellsEBVtransformedlymphocytes | 5.92 | 5.87 | 5.98 |
| CellsTransformedfibroblasts | 9.17 | 9.13 | 9.22 |
| ColonSigmoid | 7.28 | 7.23 | 7.34 |
| ColonTransverse | 3.64 | 3.60 | 3.69 |
| EsophagusGastroesophagealJunction | 7.10 | 7.04 | 7.16 |
| EsophagusMucosa | 6.37 | 6.30 | 6.44 |
| EsophagusMuscularis | 8.11 | 8.03 | 8.18 |
| HeartAtrialAppendage | 11.98 | 11.90 | 12.07 |
| HeartLeftVentricle | 7.29 | 7.21 | 7.36 |
| Liver | 2.99 | 2.94 | 3.04 |
| Lung | 7.93 | 7.86 | 7.99 |
| MuscleSkeletal | 4.27 | 4.23 | 4.31 |
| NerveTibial | 6.70 | 6.63 | 6.77 |
| Ovary | 5.39 | 5.33 | 5.45 |
| Pancreas | 11.07 | 11.00 | 11.15 |
| Pituitary | 4.67 | 4.62 | 4.71 |
| Prostate | 13.57 | 13.51 | 13.64 |
| SkinNotSunExposedSuprapubic | 7.43 | 7.37 | 7.48 |
| SkinSunExposedLowerleg | 4.06 | 4.00 | 4.12 |
| SmallIntestineTerminalIleum | 2.87 | 2.82 | 2.92 |
| Spleen | 3.70 | 3.63 | 3.76 |
| Stomach | 1.55 | 1.51 | 1.58 |
| Testis | 7.36 | 7.31 | 7.40 |
| Thyroid | 5.87 | 5.80 | 5.93 |
| Uterus | 6.54 | 6.50 | 6.58 |
| Vagina | 7.34 | 7.27 | 7.41 |
| WholeBlood | 9.56 | 9.50 | 9.62 |

|  |  |  |  |
| --- | --- | --- | --- |
|  |  |  |  |
