# Supplementary material for: A general framework for predicting the transcriptomic consequences of non-coding variation and small molecules
Source: PLoS Comput Biol. 2022 Apr 14;18(4):e1010028. doi: 10.1371/journal.pcbi.1010028 (PMC9041867; doi:10.1371/journal.pcbi.1010028)
Supplement: S2 Table — (DOCX) [file pcbi.1010028.s002.docx]

**Table S2. Tabulated summary of all tasks used to assess peaBrain performance, for both Stage 1 and Stage 2 models.**

| **Task** | **peaBrain** | **Description** |  | **Methods**  **(dataset)** | **Results** |
| --- | --- | --- | --- | --- | --- |
| A | Stage 1 | Assess Predictive capacity of the non-coding metric to identify positions with non-zero incidence of cancer-associated somatic mutations in the core promoter regions. |  | CADD, Eigen  (COSMIC) | Table 1 |
| B | Stage 1 | Assess predictive capacity of the non-coding metric to identify positions with recurrent cancer-associated somatic mutations, among all positions with at least one somatic mutation. |  | CADD, Eigen  (COSMIC) | Table 1 |
| C | Stage 1 | Assess the predictive capacity of the non-coding metric to identify variants within the 4kbps core promoter with allele-specific binding (for a subset of positions for which data was available). |  | CADD, Eigen, DeepSEA, DeepBIND, GERV, gkmSVM | Table 1 & Supplemental Note 1 |
| D | Stage 1 | Investigate how tissue-specific scores can be identify functional tissues associated with GWAS signal from complex traits |  | RTC (eQTL)-based method | Supplemental Tables 3 & 4 |
| E | Stage 2 | Compare predictive performance of peaBrain to regularized linear model |  | Elastic net | Supplemental Table 5 |
| F | Stage 2 | Assess correlation of variant estimates (for the 113 “captured” genes) with coefficients from univariately-significant eQTLS in two different populations |  | DeepSEA, MPRA, BiT-STARR-seq  (GTEx, Geuvadis) | Supplemental Figures 2-4 & Supplemental Note 2 |
| G | Stage 2 | Assess predictive capacity of peaBrain estimates to delineate variants enriched in transcriptionally-active chromatin and depleted from quiescent/repressed chromatin states |  | MPRA, BiT-STARR-seq, HiDRA  (Roadmap) | Table 2 |
| H | Stage 2 | Assess predictive capacity of peaBrain estimates to delineate variants with established regulatory function. |  | MPRA, BiT-STARR-seq, HiDRA  (RegulomeDB) | Table 3 |
