# Supplementary material for: A general framework for predicting the transcriptomic consequences of non-coding variation and small molecules
Source: PLoS Comput Biol. 2022 Apr 14;18(4):e1010028. doi: 10.1371/journal.pcbi.1010028 (PMC9041867; doi:10.1371/journal.pcbi.1010028)
Supplement: S3 Table — peaBrain p-values have been Bonferroni-corrected for multiple testing; results for all tissues are available in S4 Table. Nominal p-values are shown for the RTC (eQTL)-methodology; obtained from S8 Table of the corresponding manuscript [39]. Across all tested traits, the peaBrain framework identifies more relevant functional tissues per trait than the RTC-based method. Abbreviations: LDL, low-density lipoprotein; HDL, high-density lipoprotein; RTC, regulatory trait concordance. (DOCX) [file pcbi.1010028.s003.docx]

**Table S3.** Tabulated p-values for the top five putatively functional tissues per trait (ranked in ascending order by p-value), as predicted by the peaBrain framework and the RTC (eQTL)-based methodology (**Task D**). peaBrain p-values have been Bonferroni-corrected for multiple testing; results for all tissues are available in **Table S4**. Nominal p-values are shown for the RTC (eQTL)-methodology; obtained from Supplemental Table 8 of the corresponding manuscript[1]. Across all tested traits, the peaBrain framework identifies more relevant functional tissues per trait than the RTC-based method. **Abbreviations:** LDL, low-density lipoprotein; HDL, high-density lipoprotein; RTC, regulatory trait concordance.

|  | |  | | **peaBrain** | | **RTC (eQTL)-based** | |
| --- | --- | --- | --- | --- | --- | --- | --- |
|  | Rank | | **Tissue** | | **adjusted p** | **Tissue** | **nominal p** |
| LDL | 1 | | CellsEBVtransformedlymphocytes | | 1.81 x10^-8^ | SkinSunExposedLowerleg | 1.58 x10^-17^ |
|  | 2 | | AdiposeVisceralOmentum | | 2.54 x10^-8^ | Pancreas | 1.44 x10^-9^ |
|  | 3 | | CellsTransformedfibroblasts | | 3.45 x10^-8^ | CellsTransformedfibroblasts | 6.38 x10^-9^ |
|  | 4 | | Liver | | 4.01 x10^-8^ | NerveTibial | 1.18 x10^-8^ |
|  | 5 | | SmallIntestineTerminalIleum | | 5.06 x10^-8^ | BrainCerebellarHemisphere | 1.65 x10^-8^ |
| HDL | 1 | | ArteryTibial | | 9.46 x10^-8^ | NerveTibial | 2.36 x10^-18^ |
|  | 2 | | Stomach | | 4.46 x10^-8^ | AdiposeSubcutaneous | 8.16 x10^-16^ |
|  | 3 | | Liver | | 7.37 x10^-8^ | CellsTransformedfibroblasts | 5.41 x10^-15^ |
|  | 4 | | SmallIntestineTerminalIleum | | 8.41 x10^-8^ | SkinSunExposedLowerleg | 3.54 x10^-15^ |
|  | 5 | | AdiposeVisceralOmentum | | 1.07 x10^-7^ | SkinNotSunExposedSuprapubic | 6.39 x10^-14^ |
| Total Cholesterol | 1 | | Liver | | 4.73 x10^-11^ | SkinSunExposedLowerleg | 5.38 x10^-25^ |
|  | 2 | | CellsTransformedfibroblasts | | 5.07 x10^-11^ | Liver | 2.05 x10^-13^ |
|  | 3 | | AdiposeVisceralOmentum | | 8.33 x10^-10^ | Pancreas | 3.83 x10^-13^ |
|  | 4 | | CellsEBVtransformedlymphocytes | | 2.02 x10^-9^ | Thyroid | 9.85 x10^-13^ |
|  | 5 | | SmallIntestineTerminalIleum | | 2.99 x10^-9^ | SkinNotSunExposedSuprapubic | 5.70 x10^-12^ |
| Triglycerides | 1 | | Spleen | | 3.98 x10^-4^ | HeartLeftVentricle | 1.64 x10^-21^ |
|  | 2 | | AdrenalGland | | 8.78 x10^-4^ | Thyroid | 4.25 x10^-21^ |
|  | 3 | | CellsEBVtransformedlymphocytes | | 1.67 x10^-3^ | SkinSunExposedLowerleg | 1.52 x10^-20^ |
|  | 4 | | ArteryCoronary | | 1.63 x10^-3^ | Lung | 8.04 x10^-19^ |
|  | 5 | | AdiposeVisceralOmentum | | 1.69 x10^-3^ | AdiposeSubcutaneous | 1.15 x10^-17^ |
|  | |  | |  |  |  |  |

1. Ongen H, Brown AA, Delaneau O, Panousis NI, Nica AC, Dermitzakis ET, et al. Estimating the causal tissues for complex traits and diseases. Nature genetics. 2017;49(12):1676.
