# Supplementary material for: A general framework for predicting the transcriptomic consequences of non-coding variation and small molecules
Source: PLoS Comput Biol. 2022 Apr 14;18(4):e1010028. doi: 10.1371/journal.pcbi.1010028 (PMC9041867; doi:10.1371/journal.pcbi.1010028)
Supplement: S6 Table — The number of channels, r, is determined by the number of epigenetic and genomic annotations included in the model (minimum of 4 corresponding to the 4 DNA letter channels in class A models). The Stage 1 class B models have 32 channels, corresponding to 4 DNA sequence channels and 28 annotation channels (see Methods for details). (DOCX) [file pcbi.1010028.s006.docx]

**Table S6. Schematic of the Stage 1 peaBrain model.** The number of channels, r, is determined by the number of epigenetic and genomic annotations included in the model (minimum of 4 corresponding to the 4 DNA letter channels in class A models). The Stage 1 class B models have 32 channels, corresponding to 4 DNA sequence channels and 28 annotation channels (see **Methods** for details).

| Input Sequence:  4000 x r channels |
| --- |
| 1^st^ Convolutional Layer:  Number of Filters = 11  Size of Filters = 5  Stride = 1, Pad = 2  Leaky Rectify Activation |
| 1^st^ Pooling Layer:  Pool Size = 5, Pad = 1 |
| 2^nd^ Convolutional Layer:  Number of Filters = 11  Size of Filters = 5  Stride = 1, Pad = 2  Leaky Rectify Activation |
| 2^nd^ Pooling Layer:  Pool Size = 5, Pad = 1 |
| 3^rd^ Convolutional Layer:  Number of Filters = 11  Size of Filters = 5  Stride = 1, Pad = 2  Leaky Rectify Activation |
| 3^rd^ Pooling Layer:  Pool Size = 5, Pad = 1 |
| Dropout Layer:  p = 0.5 |
| Dense Fully-Connected Layer:  Number of Units = 1001  Linear Activation |
| Output Layer:  Number of Units = 1  Linear Activation |
| Output Value:  Average Gene Abundance |
