# Supplementary material for: A general framework for predicting the transcriptomic consequences of non-coding variation and small molecules
Source: PLoS Comput Biol. 2022 Apr 14;18(4):e1010028. doi: 10.1371/journal.pcbi.1010028 (PMC9041867; doi:10.1371/journal.pcbi.1010028)
Supplement: S7 Table — Values in red denote layers with differing values between the three networks. The network for the centre split is identical to the Stage 1 peaBrain model for the core promoter region; the networks for the upstream and downstream splits are identical to the Stage 1 peaBrain model for distal sequences. Thus, Stage 2 peaBrain can be thought of as a consolidation of the separate Stage 1 models. (DOCX) [file pcbi.1010028.s007.docx]

**Table S7. Schematic of the Stage 2 peaBrain model, which is composed of three separate networks connected by a dense layer prior to prediction.** Values in red denote layers with differing values between the three networks. The network for the centre split is identical to the Stage 1 peaBrain model for the core promoter region; the networks for the upstream and downstream splits are identical to the Stage 1 peaBrain model for distal sequences. Thus, Stage 2 peaBrain can be thought of as a consolidation of the separate Stage 1 models.

|  | Input Sequence  1Mbps x 4 channels |  |
| --- | --- | --- |
| Upstream Split  0.498Mbps x 4 channels | **Centre Split**  4kbps x 4 channels | **Downstream Split**  0.498Mbps x 4 channels |
| 1^st^ Convolutional Layer:  Number of Filters = 11  Size of Filters = 5  Stride = 1, Pad = 2  Leaky Rectify Activation | **1^st^ Convolutional Layer:**  Number of Filters = 11  Size of Filters = 5  Stride = 1, Pad = 2  Leaky Rectify Activation | **1^st^ Convolutional Layer:**  Number of Filters = 11  Size of Filters = 5  Stride = 1, Pad = 2  Leaky Rectify Activation |
| 1^st^ Pooling Layer:  Pool Size = 100, Pad = 1 | **1^st^ Pooling Layer:**  Pool Size = 5, Pad = 1 | **1^st^ Pooling Layer:**  Pool Size = 100, Pad = 1 |
| 2^nd^ Convolutional Layer:  Number of Filters = 11  Size of Filters = 5  Stride = 1, Pad = 2  Leaky Rectify Activation | **2^nd^ Convolutional Layer:**  Number of Filters = 11  Size of Filters = 5  Stride = 1, Pad = 2  Leaky Rectify Activation | **2^nd^ Convolutional Layer:**  Number of Filters = 11  Size of Filters = 5  Stride = 1, Pad = 2  Leaky Rectify Activation |
| 2^nd^ Pooling Layer:  Pool Size = 50, Pad = 1 | **2^nd^ Pooling Layer:**  Pool Size = 5, Pad = 1 | **2^nd^ Pooling Layer:**  Pool Size = 50, Pad = 1 |
| 3^rd^ Convolutional Layer:  Number of Filters = 11  Size of Filters = 5  Stride = 1, Pad = 2  Leaky Rectify Activation | **3^rd^ Convolutional Layer:**  Number of Filters = 11  Size of Filters = 5  Stride = 1, Pad = 2  Leaky Rectify Activation | **3^rd^ Convolutional Layer:**  Number of Filters = 11  Size of Filters = 5  Stride = 1, Pad = 2  Leaky Rectify Activation |
| 3^rd^ Pooling Layer:  Pool Size = 10, Pad = 1 | **3^rd^ Pooling Layer:**  Pool Size = 5, Pad = 1 | **3^rd^ Pooling Layer:**  Pool Size = 10, Pad = 1 |
| Dropout Layer:  p = 0.5 | **Dropout Layer:**  p = 0.5 | **Dropout Layer:**  p = 0.5 |
| Dense Fully-Connected Layer:  Number of Units = 50  Linear Activation | **Dense Fully-Connected Layer:**  Number of Units = 50  Linear Activation | **Dense Fully-Connected Layer:**  Number of Units = 50  Linear Activation |
|  | **Dense Fully-Connected Layer:**  Number of Units = 50  Linear Activation |  |
|  | **Output Layer:**  Number of Units = 1  Linear Activation |  |
|  | **Output Value:**  Individual Gene Abundance |  |
