# Supplementary figures and images for: A general framework for predicting the transcriptomic consequences of non-coding variation and small molecules
Source: PLoS Comput Biol. 2022 Apr 14;18(4):e1010028. doi: 10.1371/journal.pcbi.1010028 (PMC9041867; doi:10.1371/journal.pcbi.1010028)

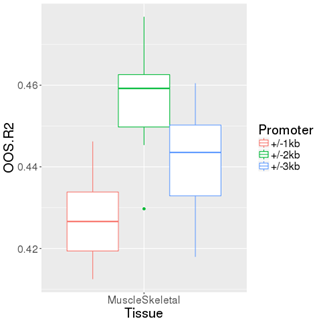

Supplement: S1 Fig — (PNG) [file pcbi.1010028.s008.png]

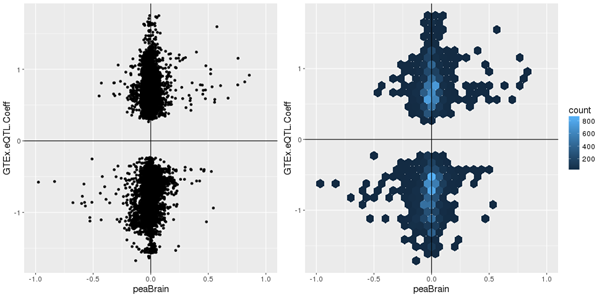

Supplement: S2 Fig — Each point corresponds to a variant that is univariately significant in the GTEx eQTL analysis (n = 16,019 eQTLs). The y-axis is the magnitude of the univariate GTEx eQTL coefficient for the corresponding variant. The correlation between the GTEx coefficient and the peaBrain prediction is positive and significant (Spearman’s rho = 0.09; p = 3.02 x10^-32^). (PNG) [file pcbi.1010028.s009.png]

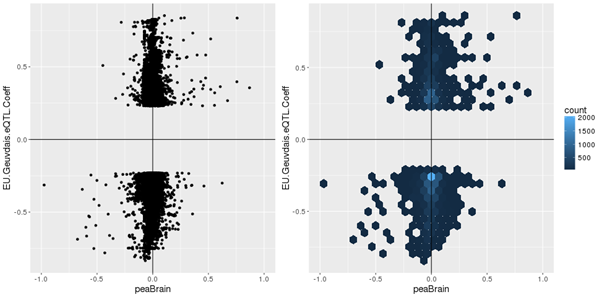

Supplement: S3 Fig — Each point corresponds to a variant that is univariately significant in the EU-Geuvadis eQTL analysis (n = 17,279 eQTLs). The y-axis is the magnitude of the univariate EU-Geuvadis eQTL coefficient for the corresponding variant. The correlation between the EU-Geuvadis coefficient and the peaBrain prediction is positive and significant (Spearman’s rho = 0.10; p = 9.60 x10^-38^). (PNG) [file pcbi.1010028.s010.png]

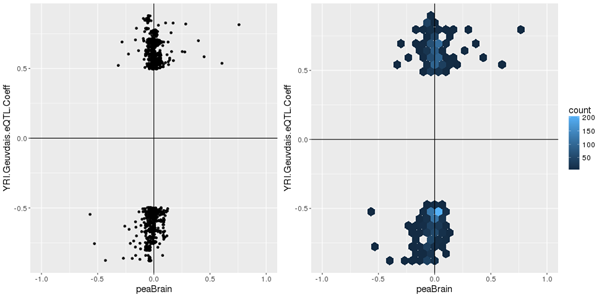

Supplement: S4 Fig — Each point corresponds to a variant that is univariately significant in the YRI-Geuvadis eQTL analysis (n = 1601 eQTLs). The y-axis is the magnitude of the univariate YRI-Geuvadis eQTL coefficient for the corresponding variant. The correlation between the YRI-Geuvadis coefficient and the peaBrain prediction is positive and significant (Spearman’s rho = 0.18; p = 8.64 x10^-13^). (PNG) [file pcbi.1010028.s011.png]
