# Supplementary material for: A general framework for predicting the transcriptomic consequences of non-coding variation and small molecules
Source: PLoS Comput Biol. 2022 Apr 14;18(4):e1010028. doi: 10.1371/journal.pcbi.1010028 (PMC9041867; doi:10.1371/journal.pcbi.1010028)
Supplement: S1 Text — The note also highlights how activations of the penultimate layer of the peaBrain model can be used as a continuous and compressed representation (i.e. embedding) of genes. These embeddings, or equivalently, neural activations, capture both the annotated DNA (input) and its additive contributions to tissue-specific abundance (output) in a compressed form amenable to downstream analyses (such as network-based analyses). These embeddings display interesting properties, including the encoding of correlation information and membership to pathways/curated gene sets. Importantly, these embeddings are in a linear space, such that the pairwise cosine similarity between these dense gene representations is proportional to the measured RNA-seq correlation between the gene pair. In other words, co-regulation and co-expression may be discovered by leveraging linear structure within the embeddings (e.g., adding embeddings of two genes to discover their co-expression with a third). (DOCX) [file pcbi.1010028.s012.docx]

**S1 Text**

**Regularized linear models are poor models for predicting mean abundance from core promoter sequences (10-fold cross-validated average cv-r^2^ < 0).** To assess how simple linear models compare to “deep learning” models, we fitted linear models by minimizing a regularized empirical squared loss, with a L2 (squared euclidean norm) penalty, using stochastic gradient descent from python’s sci-kit learn (sklearn). The input of the class B models (non-specific annotated DNA model; 1D matrix with 32 channels) was flattened prior to modelling, i.e. the mean abundance each gene was modelled as a linear function of 128,000 variables (most were binary variables). For brevity, we limited our analysis to skeletal muscle, the tissue with the largest number of samples in GTEx. The model was fit using the partial fit function from SGDRegressor with sklearn using default parameters; the same exit criteria as described in **Methods** were applied. We noted that 10-fold cross-validated average cv-r^2^ was consistently below zero, with performance considerably worse than the peaBrain model. The gap in performance was considerable (precludes visualization). We also repeated this analysis using a single dense neural network layer with linear activations, and noted that the 10-fold cross-validated average cv-r^2^ was below 0; a single dense layer with linear activations is equivalent to a simple linear model.

**Fully connected neural networks are slower and more memory-intensive, with slightly worse performance than convolutional neural networks; non-linear activations for convolutional layers improve performance over linear activations.** We compared fully connected dense neural networks to the convolutional neural networks at the heart of peaBrain (**Fig A**) for class B skeletal muscle model. We replaced each pair of convolutional-pooling layers with a single dense layer; the number of neurons in the three dense layers was limited only by GPU memory. The penultimate layer and single output neuron were kept consistent between the models. Using the skeletal muscle Class B input, we noted that fully connected performed slightly worse (cv-r^2^ = 0.42) than the convolutional neural networks (cv-r^2^ = 0.46). Increasing the number of layers allows fully connected neural network to reach performance parity with CNNs, at the cost of increased memory and computational cost (not feasible for Stage 2 peaBrain analyses). We subsequently wanted to assess the importance of the non-linear activation for the convolutional layers of peaBrain. We constructed an identical model, but replacing all CNN activations with linear functions and noted that performance for this model was consistently worse (cv-r^2^ = 0.43) than the classical peaBrain architecture (cv-r^2^ = 0.46). It is important to note that this is not an exhaustive search of the ideal set of parameters, but an exploratory analysis to begin to understand peaBrain’s performance.

**DNA sequence, annotated with experimentally-derived TFBS, from core promoter sequences are insufficient to predict mean abundance with high accuracy** – **epigenetic/histone markers contain the bulk of the information and are not readily accessible from the DNA sequence alone.** We were interested in determining the contribution of epigenetic/histone makers, alongside more general genomic annotations (such as coding sequences), in predicting the mean abundance of genes. In particular, we wanted to explore whether the DNA sequence alone was sufficient to predict expression in skeletal muscle. We noted that increasing the number of convolutional layers or the number of filters did not improve model performance (**Fig A**). Explicitly incorporating TFBS into the model (i.e. annotating the DNA only and explicitly with TFBS) only improved performance slightly (cv-r^2^ = 23%), and was still considerably worse than the full class B model with epigenetic/histone marker annotations (cv-r^2^ = 46%; **Fig A**). (Class-A DNA-only models had an average cv-r^2^ of 16% for skeletal muscle; class-C models annotated with tissue-specific information had an average cv-r^2^ of 57%.) The TFBS were collected from the Gene Transcription Regulation Database (GTRD) v17.4 with data on 476 human transcription factors and included peak calling with four different software (MACS, SISSRs, GEM, and PICS). In addition to including the processed peak calls, we also incorporated clusters (i.e. peaks merged for the same transcription factor but under different experimental conditions) and meta-clusters (i.e. non-redundant peaks synthesized from all four methods). This absence of improvement suggests that peaBrain model already recognizes many of the TFBS; identified by the convolutional filters inherent to the model architecture. These results indicate that experimentally-derived epigenetic and genomic annotations add information to that contained in the DNA sequence alone. As described in the main text, this is broadly consistent with the observation that other convolutional neural networks models like DeepSEA are better at predicting TFBS (median AUC = 0.958) than at predicting histone modifications (median AUC = 0.856) (Zhou and Troyanskaya 2015).

**Neural activations of penultimate layer of peaBrain model can be used to construct an embedding from the genes that encodes correlation information.**  Having demonstrated the predicative ability of the peaBrain model (see **Main text** for details), we were subsequently interested in using the activations from the penultimate layer of the model as a continuous (and compressed) representation of the genes. These neural activations capture both the annotated DNA (input) and its additive contributions to tissue- and phenotype-specific abundance (output) in a compressed form amenable to downstream analyses. Furthermore, as these vectors were obtained from a regression model, they readily capture only the salient portions of DNA abundance encoded in the annotated-genome (the weights of the model corresponding to the transcription factor that regulate and interact with this genome). Because of model choice, the mean abundance of each gene was encoded as a linear combination of the vector elements, *i.e.* the output of the regression model. As with our earlier analyses, for brevity, we limited our analysis of the properties of the embeddings to class B models for skeletal muscle. We observed, for the skeletal muscle embeddings, that pairwise cosine similarity between these dense gene representations corresponded to the measured RNAseq correlation between the gene pair. After excluding self-correlations and weakly correlated genes (RNAseq rho < 0.5), we noted that the cosine similarity of the embedding was significantly correlated (Spearman’s rho = 0.18; p < 2.2 x10^-16^) to the experimentally RNAseq-derived correlation. This suggests the annotated-DNA model, without supervision, imposes a linear structure on this vector space: the angle between the vectors corresponds to the co-regulation of the gene pair.

**peaBrain-derived gene embeddings also encode membership to pathways and other curated gene sets.** We were interested in further exploring the utility of these embeddings in other applications. We noted that this dense representation from the class B skeletal muscle pea Brain model encodes membership to the MSigDB Hallmark curated gene sets (average 10-fold cross-validated for all pathways AUC = ~0.70, **Table A**), suggesting that the representations themselves, not only encode abundance and regulatory information, but also functional relationships. (We filtered pathway sets not relevant to the tissues, such as “PANCREAS_BETA_CELLS”, “COMPLEMENT”, or “SPERMATOGENSIS”). Taken all together, this suggests the gene embeddings capture both the annotated DNA (input) and its additive contributions to tissue-specific abundance (output) in a compressed form amenable to downstream analyses (e.g. network-based analyses).

**Table A.**  Tabulated 10-fold cross-validated AUC for genomewide pathway membership predictions using class B MuscleSkeletal Embeddings.

| **Hallmark Gene Set** | **10-fold cross-validated**  **average auc** |
| --- | --- |
| MYC_TARGETS_V1 | 0.80 |
| MYC_TARGETS_V2 | 0.79 |
| G2M_CHECKPOINT | 0.77 |
| UNFOLDED_PROTEIN_RESPONSE | 0.76 |
| OXIDATIVE_PHOSPHORYLATION | 0.76 |
| MTORC1_SIGNALING | 0.76 |
| EPITHELIAL_MESENCHYMAL_TRANSITION | 0.74 |
| MITOTIC_SPINDLE | 0.74 |
| E2F_TARGETS | 0.73 |
| REACTIVE_OXIGEN_SPECIES_PATHWAY | 0.73 |
| TNFA_SIGNALING_VIA_NFKB | 0.72 |
| PROTEIN_SECRETION | 0.72 |
| TGF_BETA_SIGNALING | 0.71 |
| UV_RESPONSE_DN | 0.71 |
| PI3K_AKT_MTOR_SIGNALING | 0.71 |
| DNA_REPAIR | 0.71 |
| HYPOXIA | 0.70 |
| P53_PATHWAY | 0.69 |
| APOPTOSIS | 0.68 |
| APICAL_JUNCTION | 0.68 |
| ADIPOGENESIS | 0.67 |
| MYOGENESIS | 0.66 |
| IL2_STAT5_SIGNALING | 0.66 |
| ANGIOGENESIS | 0.66 |
| GLYCOLYSIS | 0.65 |
| PANCREAS_BETA_CELLS | 0.65 |
| ANDROGEN_RESPONSE | 0.65 |
| KRAS_SIGNALING_DN | 0.64 |
| HEME_METABOLISM | 0.64 |
| CHOLESTEROL_HOMEOSTASIS | 0.64 |
| HEDGEHOG_SIGNALING | 0.63 |
| APICAL_SURFACE | 0.63 |
| UV_RESPONSE_UP | 0.63 |
| INTERFERON_GAMMA_RESPONSE | 0.63 |
| ESTROGEN_RESPONSE_EARLY | 0.62 |
| INTERFERON_ALPHA_RESPONSE | 0.62 |
| ESTROGEN_RESPONSE_LATE | 0.61 |
| NOTCH_SIGNALING | 0.61 |
| KRAS_SIGNALING_UP | 0.61 |
| INFLAMMATORY_RESPONSE | 0.61 |
| COAGULATION | 0.60 |
| SPERMATOGENESIS | 0.60 |
| WNT_BETA_CATENIN_SIGNALING | 0.58 |
| PEROXISOME | 0.58 |
| IL6_JAK_STAT3_SIGNALING | 0.57 |
| ALLOGRAFT_REJECTION | 0.57 |
| COMPLEMENT | 0.57 |
| BILE_ACID_METABOLISM | 0.56 |
| XENOBIOTIC_METABOLISM | 0.56 |
| FATTY_ACID_METABOLISM | 0.56 |

**Figure A.** **Boxplots of 10-fold cross-validated cv-r^2^, as assessed in skeletal muscle.** Performance as assessed for class A models (labelled as “class A peaBrain – DNA only”), class A with TFBS annotations (labelled as “class A peaBrain – DNA+TFBS”), class B models with tissue-agnostic annotations (“class B peaBrain – CNNs”), fully connected neural networks (“class B – fully-connected”), class B models with linear activation functions (“class B peaBrain – linear activations”), class B models with increased number of layers (“class B peaBrain – more layers”), class B models with increased number of filters (“class B peaBrain – more filters”), and class C models with tissue-specific annotations (“class C peaBrain”).


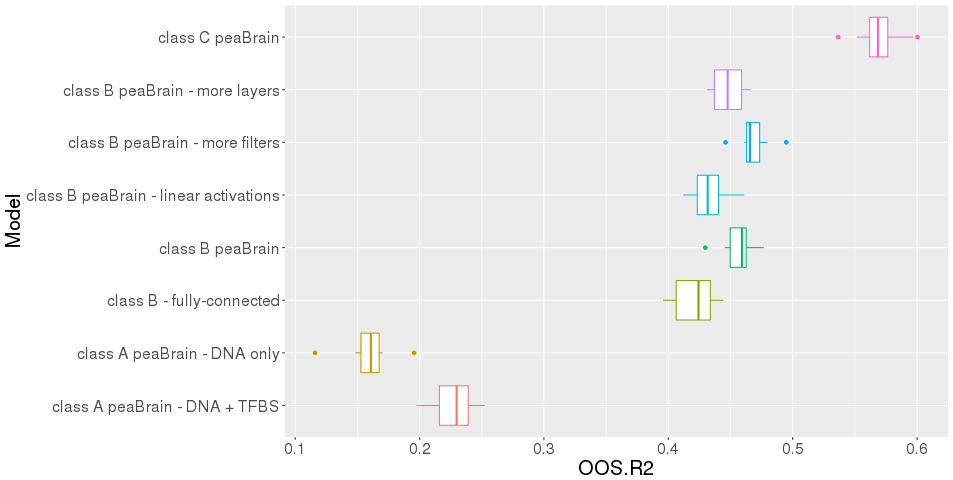


**Figure B.** Rank correlation plot for TF-binding algorithms and the peaBrain impact score. JASPAR, MEME_1 and MEME_2 are PWM-approaches.


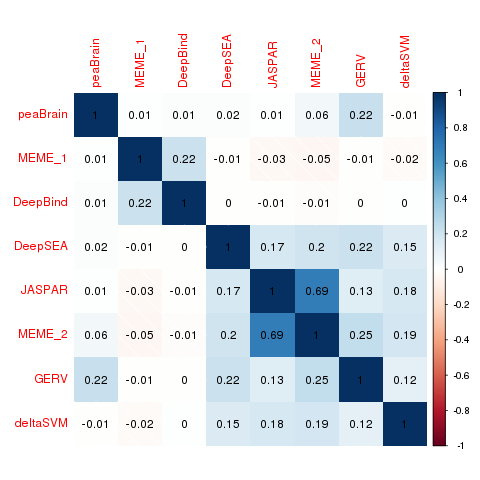


**REFERENCES**

Alipanahi B, Delong A, Weirauch MT, Frey BJ. 2015. Predicting the sequence specificities of DNA-and RNA-binding proteins by deep learning. *Nature biotechnology* **33**: 831.

Lee D, Gorkin DU, Baker M, Strober BJ, Asoni AL, McCallion AS, Beer MA. 2015. A method to predict the impact of regulatory variants from DNA sequence. *Nature genetics* **47**: 955.

Wagih O, Merico D, Delong A, Frey BJ. 2018. Allele-specific transcription factor binding as a benchmark for assessing variant impact predictors. *bioRxiv*: 253427.

Zeng H, Hashimoto T, Kang DD, Gifford DK. 2015. GERV: a statistical method for generative evaluation of regulatory variants for transcription factor binding. *Bioinformatics* **32**: 490-496.

Zhou J, Troyanskaya OG. 2015. Predicting effects of noncoding variants with deep learning–based sequence model. *Nature methods* **12**: 931.
