# Supplementary material for: A general framework for predicting the transcriptomic consequences of non-coding variation and small molecules
Source: PLoS Comput Biol. 2022 Apr 14;18(4):e1010028. doi: 10.1371/journal.pcbi.1010028 (PMC9041867; doi:10.1371/journal.pcbi.1010028)
Supplement: S2 Text — By modelling the transitions between consecutive neural progenitor cell stages, we identified the subset of genes in each stage-specific differentiation whose expression is directly altered by epigenetic modifications in their promoter sequences. Among the genes identified in the differentiation of neuro-epithelial and mid-radial glial cells, we note significant enrichment of genes implicated in schizophrenia, autism, bipolar, and depression (1.5–2.6 fold enrichment; Fisher’s exact p < 0.05); a trend that becomes more pronounced when limited to genes implicated using genetic associations alone. With nearly 5–10% labelled as transcription factors (1.53–3.24 fold enrichment), this cross-disease enrichment provides a putatively causal mechanism early in neural development for the shared genetic correlation between these psychiatric phenotypes–an observation that is difficult to extract from GWAS data alone and is missed by differential peak/gene expression analyses. (DOCX) [file pcbi.1010028.s013.docx]

**S2 Text**

High-throughput assays (HTAs) are the cornerstone of modern drug discovery and a useful tool to translating the hundreds of genetic discoveries associated with human traits and disease into functional understanding(Kalita et al., 2018; Melnikov et al., 2012; Subramanian et al., 2017; Tewhey et al., 2016; Wang et al., 2017). All high-throughput assays can be described as empirical assessments of the activity of biological entities (e.g. genetic variation, DNA sequences, small molecules) by a standardized output, usually in the form of optically detectable labels (i.e. reporters), or more rarely, using (scalable) high-dimensional measurements (e.g. L1000, RNA-seq). Increasingly, with mounting evidence suggesting that the causal variants at genetic loci affect non-regulatory changes (rather than protein-coding alterations)(Hindorff et al., 2009), many of these HTAs – including those used in drug discovery(Subramanian et al., 2017) – are focussed on the immediate impact of biological entities on the transcriptome. In fact, these expression-centred HTAs can be described generally as:

biological entity (e.g. DNA sequence or small molecule) **→** measured expression output

Most, if not all, current research has focussed on the development of these experimental methods and the analytic techniques necessary to analyse the generated data(Kalita et al., 2018; Subramanian et al., 2017; Wang et al., 2017). Here, we introduce a general, modular in silico framework – **p**romoter-and-**e**nhancer-derived **a**bundance (**pea**Brain) framework – with the goal of recapitulating these expression-centred HTAs as computational models on graphical processing units (GPUs) to enable inexpensive and more scalable interrogations of the functional impact of diverse biological entities on expression. The convolutional neural network architecture at the core of this framework leverages promoter sequences to emulate the transcriptional machinery of cell lines/tissues, allowing us to re-create (**Fig A**, on next page): (a) DNA sequence reporter assays, (b) small molecule high-throughput screens, and (c) shRNA high-throughput screens; and to (d) jointly model whole genome sequences to identify putatively functional eQTLs – all using small extensions of the same architecture and model hyperparameters. These large-scale, high-throughput interrogations provide us with compelling biological hypotheses, including a putatively causal mechanism that accounts for the genetic correlation between psychiatric illness, a new approach to drug repositioning, and hundreds of new regulatory interactions with experimental support.


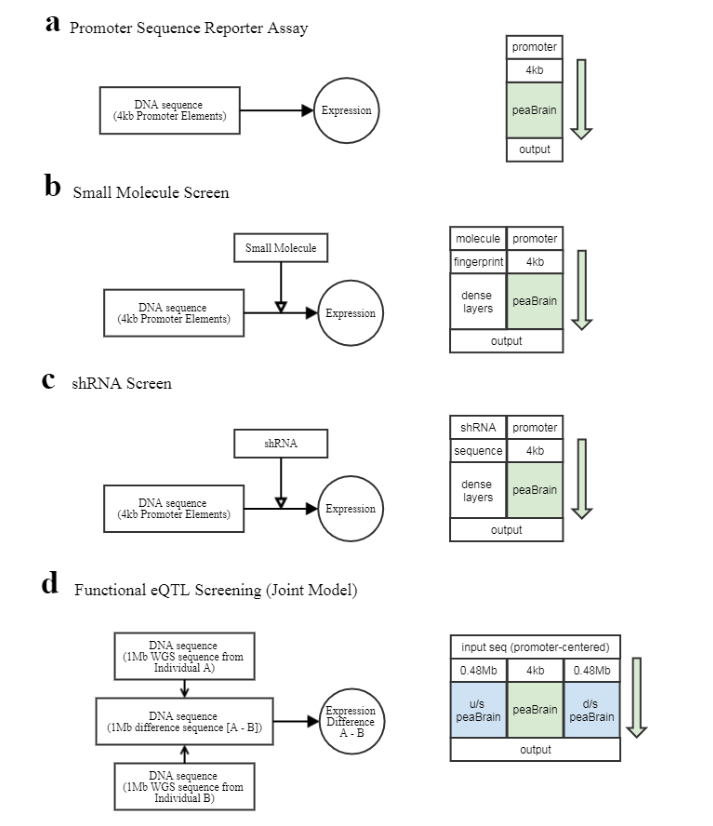


**Figure A.** An overview of the biological architecture underlying the modular extensions and applications of the peaBrain framework. **(a)** The DNA sequence reporter assay is at the core of the peaBrain framework, which can be extended to incorporate **(b)** molecular fingerprints to model the impact of small molecules on expression or **(c)** shRNA oligo-sequences to model the impact of RNA interference on expression. **(d)** If we train the peaBrain model on larger sequences (centred on the promoter), we can jointly model the impact of genotypic variation (substitutions, insertions, and deletions) to predict individual expression and identify putatively functional eQTLs. Abbreviations: u/s, upstream; d/s, downstream.

**peaBrain DNA reporter model can predict the transcriptomic consequences of epigenetic modifications along the neural differentiation trajectory.**

Having established the predictive capacity of peaBrain, we were interested in exploring the utility of the model in understanding the consequences of epigenetic modifications that underlie the key cell fate decisions that occur during normal development/differentiation. (Class B and class C models incorporate epigenomic annotations alongside DNA promoter sequence as input to the algorithm.) As a case study, we focussed on neural induction of human pluripotent stem cells (WA9/H9 cell line) during four consecutive stages for which data was available: human embryonic stem cells, neuroepithelial cells, early radial glial cells, and mid radial glial cells. We constructed a peaBrain model specific to each stage that predicts stage-specific expression from the DNA promoter sequence annotated with H3K4me1, H3K4me3, H3K27ac, and H3K27me3 as well as DNA methylation peaks (from the corresponding stage). Each stage-specific peaBrain model (n = 4) emulates the transcriptional machinery active), which we can use to identify the subset of genes between any two consecutive stages whose expression is directly altered by epigenetic modifications in the core promoter elements (see **Methods**). From all genes with non-zero expression in at least one of the four stages (n = 29,696), between embryonic and neuroepithelial cells, we identified 1271 genes whose normalized expression changed by more than 0.5 standard deviations as a consequence of epigenetic modifications (at a q-value < 0.05). Similarly, the expression profiles of 555 genes were identified as transcriptionally altered (downstream of epigenetic alterations in the promoter) during the transition from neuroepithelial to early radial glial cells, and 851 genes for the transition between early and mid radial glial cells. Nearly all of these genes were undetected with simple differential expression analyses, which may be a consequence of limited power to detect with classical approaches (samples were collected in replicate only).

We note that, among the genes identified in the differentiation of neuro-epithelial (from embryonic stem cells) and mid radial glial cells (from early radial glial cells), there was significant enrichment of genes implicated in schizophrenia, autism, bipolar, and depression (1.5-2.6 fold enrichment; Fisher’s exact p < 0.05; **Tables A-C**). This trend became more pronounced and more significant when limited to genes implicated using genetic associations alone (Tables 1-3). With nearly 5-10% labelled as transcription factors (1.53-3.24 fold enrichment), this cross-disease enrichment suggests a putatively causal biological mechanism early in neural development for the shared genetic correlation between these psychiatric phenotypes – an observation that is difficult to extract from GWAS data alone and is missed by pairwise differential peak/gene expression analyses. The enrichment of disease-implicated genes was smaller and less significant for during the transition from neuroepithelial to early radial glial cells (p-value > 0.05 for bipolar and autism), implicating the shared disease processes in sub-portions of neural development.

**Table A.** We observed significant enrichment of genes implicated in psychiatric disease among the set of genes whose expression is altered by epigenetic modifications in promoter sequences during the transition from human embryonic stem to neuroepithelial cells. The fraction column denotes the percentage of the transcriptionally-altered genes that are also disease genes, e.g. 7.00% of genes whose expression is altered by epigenetic modifications during this transition are implicated in autism. We assessed enrichment using Fisher’s exact test. For all diseases, we included the corresponding gene sets from the Open Targets platform. For autism, we included two gene sets (one from Open Targets and one from Simon’s Foundation [labelled by SFARI]).


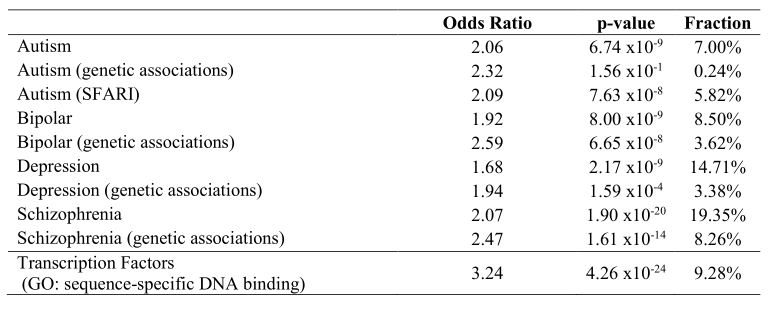


**Table B.** (*on next page*) We observed reduced enrichment of genes implicated in psychiatric disease in the set of epigenetically-driven transcriptomically-altered gene expression profiles during the transition from neuroepithelial to early radial glial cells. Conservative adjustment for multiple testing eliminates all significance.


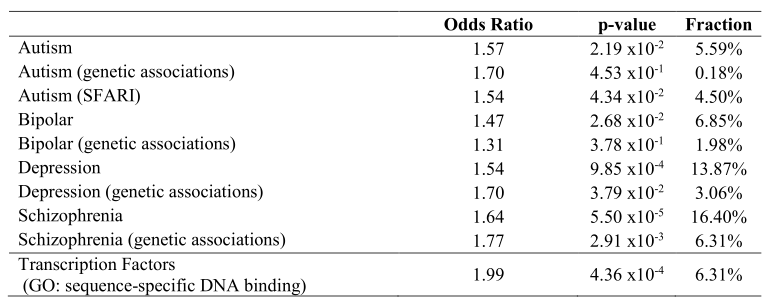


**Table C.** As observed in the differentiation of neuroepithelial cells (from embryonic stem cells), we note the enrichment of genes implicated in psychiatric disease among the set of genes whose expression is altered by epigenetic modifications during the transition from early to mid radial glial cells.


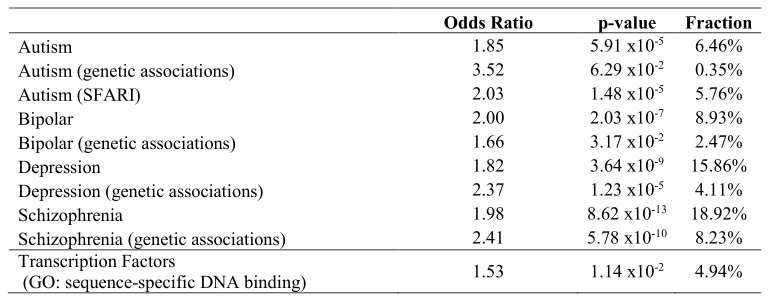


**REFERENCES**

Hindorff, L. A., Sethupathy, P., Junkins, H. A., Ramos, E. M., Mehta, J. P., Collins, F. S., & Manolio, T. A. (2009). Potential etiologic and functional implications of genome-wide association loci for human diseases and traits. *Proceedings of the National Academy of Sciences, 106*(23), 9362-9367.

Kalita, C. A., Brown, C. D., Freiman, A., Isherwood, J., Wen, X., Pique-Regi, R., & Luca, F. (2018). High throughput characterization of genetic effects on DNA:protein binding and gene transcription. *bioRxiv.* doi:10.1101/270991

Melnikov, A., Murugan, A., Zhang, X., Tesileanu, T., Wang, L., Rogov, P., . . . Kinney, J. B. (2012). Systematic dissection and optimization of inducible enhancers in human cells using a massively parallel reporter assay. *Nature biotechnology, 30*(3), 271-277.

Subramanian, A., Narayan, R., Corsello, S. M., Peck, D. D., Natoli, T. E., Lu, X., . . . Asiedu, J. K. (2017). A next generation connectivity map: L1000 platform and the first 1,000,000 profiles. *Cell, 171*(6), 1437-1452. e1417.

Tewhey, R., Kotliar, D., Park, D. S., Liu, B., Winnicki, S., Reilly, S. K., . . . Schaffner, S. F. (2016). Direct identification of hundreds of expression-modulating variants using a multiplexed reporter assay. *Cell, 165*(6), 1519-1529.

Wang, X., He, L., Goggin, S., Saadat, A., Wang, L., Claussnitzer, M., & Kellis, M. (2017). High-resolution genome-wide functional dissection of transcriptional regulatory regions in human. *bioRxiv*, 193136.
