# Supplementary material for: A general framework for predicting the transcriptomic consequences of non-coding variation and small molecules
Source: PLoS Comput Biol. 2022 Apr 14;18(4):e1010028. doi: 10.1371/journal.pcbi.1010028 (PMC9041867; doi:10.1371/journal.pcbi.1010028)
Supplement: S3 Text — Predictive performance of this small molecule screen is on par with in vitro experimental replication of external test sets, allowing us to impute expression for all clinically approved molecules in the ChEMBL database. By training on the imputed expression profiles for all molecules first approved prior to the year 2000, we are able to retrospectively identify molecules that would later be assigned to the corresponding indications (post-2000), with 64–86% increase in F1-scores (i.e., the harmonic mean of precision and recall, a measure of model performance and accuracy that is robust to class imbalance), 22–63% increase in precision, 75–94% increase in recall, and 13–19% increase in area under precision-recall curve (AUPRC), compared to current chemoinformatic/molecule structure-based approaches. (DOCX) [file pcbi.1010028.s014.docx]

**S3 Text**

**Small molecule extension of the peaBrain DNA-reporter assay captures ~84% of the variance in the average molecule perturbed gene expression profiles of L1000 landmark transcripts.**

To predict the transcriptomic perturbations of small molecules, we extended the peaBrain DNA reporter assay to incorporate molecular fingerprints (boolean arrays/bitmaps [i.e. a list of 1s and 0s] that are characteristic of the molecule structure), in addition to the DNA core promoter sequence as input (which directly accounted for copy number and genotypic variation, see **Methods**). This task demonstrates the utility of the modular structure of peaBrain and the ‘shareability’ of information that is a consequence of its architecture/design. As highlighted in **Table A,** the small molecule extension can be described as:

promoter + molecular fingerprint 🡪 expression

where the fingerprint can be thought of as the “genotype” of the molecule. Given a core promoter sequence and a molecular fingerprint, the small molecule peaBrain model will predict the expression of the corresponding gene. We limited our analysis to molecular fingerprints – encoded as a standard bit vectors – generated using Morgan’s algorithm(Rogers and Hahn 2010) from the molecular-input line-entry system (SMILES) specification for each molecule. For each molecule profile (at 10uM dosage), we rank-normalized expression (averaging the replicates) and min-max scaled (per gene) prior to training (see **Online** **Methods** for details).

**Table A.** Overview of small molecule extension and its biological applications. The peaBrain small molecule model is a simple extension of the original peaBrain DNA reporter. Internal testing procedure describes the approach by which the model was trained and assessed on the training set. We filtered the phase II LINCS dataset to exclude molecules in phase I. The phase II (external set) is used only once: to assess performance once model weights are learnt using the training set.

| **peaBrain small molecule model:** promoter + molecule fingerprint 🡪 expression | | | | |
| --- | --- | --- | --- | --- |
| *Description:* For a given cell type, small molecule, and promoter element, predict expression of gene. Simple extension of the peaBrain DNA reporter model (described above). | | | | |
| **Technical Overview**  *Objective:* Assess the predictive performance of the peaBrain small molecule model | | | | |
| **Training set** | **Internal Assessment** | **Types of Models Constructed** | **External Assessment** | **Notes** |
| GSE92742  LINCS phase I | 4-fold  Monte Carlo validation  (5% testing) | Single model: promoter DNA sequence + molecule fingerprint | GSE70138  LINCS phase II | Models constructed for single dose (10uM) |
| **Biological Applications:**  1. Drug re-positioning (can retrospectively predict approval for relevant indications)  2. Drug discovery (identify compounds that selectively inhibit cancer hallmark genes) | | | | |

Training was conducted with expression profile data downloaded from the LINCS phase I L1000 Connectivity Map (GSE92742) dataset on NCBI’s Gene Expression Omnibus (GEO; see **URLs in S4 Text**)(Subramanian et al. 2017). (The LINCS phase II [GSE70138] dataset was used as the external test set.) We limited our investigation to cell lines for which genotype, copy-number, and both phase I and phase II data was available (n = 5 cell lines: A375, A549, HT29, PC3, and MCF7). Both phase I and phase II LINCS projects use the L1000 assay, which measures the expression profile of 978 landmark transcripts (using a microarray-like technology). We discarded any imputed transcripts before training (see **Methods**). Genotype and copy-number data for all 5 cell lines included in the analysis, identified by PICNIC analysis of Affymetrix SNP6.0 array data, were obtained from the Sanger’s Institute COSMIC cell line webpage (see **URLs in S4 Text** and **Methods** for details).

We assessed the predictive performance of the cell line-specific peaBrain models using a Monte Carlo-validation scheme (**Fig A,** *on next page*). In each fold, 5% of small molecules were randomly withheld as a test set (that is, neither the fingerprint nor expression profile is ever seen by the model). Random selection of the test set was sufficient as molecular similarity was very weakly correlated with transcriptomic similarity (median Spearman’s rho = 1.0%). Of the remaining 95% of small molecules, 10% was randomly selected as validation to check for early exit criteria (i.e. not used to train the model parameters, see **Methods**). The model was then trained on the remaining drug profiles to predict the gene-level expression, 24 hours post-exposure, for any pair of molecular fingerprints and core promoter sequences (per cell line). We note that, across all cell lines, the model had a median oos-r^2^ of 84% (**Fig A**). Importantly, this performance was 12% better (on average) than simply using the expression profile of the closest structurally similar molecule in the training set (e.g. using a structurally-similar oestradiol analogue as the prediction of the oestradiol expression profile). This suggests that the model is able to extract more biologically-relevant information (likely from the promoter sequences) than that encoded in the molecular fingerprint used as input to the algorithm.


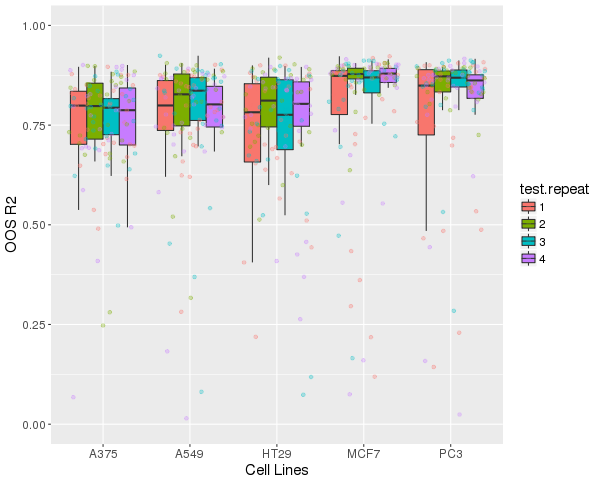


**Figure A.** Given a core promoter sequence and a molecular fingerprint, the small molecule peaBrain model can accurately the transcriptomic perturbation of the corresponding gene across all 5 cell lines. Each point represents oos-r^2^ of a single unique small molecule (included in the test set). We performed 4 folds of Monte Carlo validation per cell line (folds denoted by the colour). The y-axis was clipped at zero; only seven molecules across all cell lines and test/validation repeats had an oos-r^2^ < 0. **Abbreviations:** OOS R2, out-of-sample r^2^.

To further test the model generalizability, we assessed predictive performance on the rank-normalized LINCS phase II GSE70138 (external) dataset. Moreover, to calibrate our assessment to the upper bound of assay reproducibility, for each test set, we sub-selected molecules assessed in both the phase I and phase II LINCS experiments. This way we are able to benchmark peaBrain predictive performance to experimental replicates *(*using the same assay and platform); that is, for each small molecule, we performed two tests: how well the imputed peaBrain profile compares to the phase II profile, and how well the phase I profile (i.e. the experimental replicate) compares to phase II profile. We note almost identical performance between peaBrain predictions (median rho between peaBrain and phase II profiles = 54%) and the phase I experimental replicates in predicting phase II profiles (median rho between phase I and phase II profiles = 47%), suggesting that *in silico* peaBrain predictions of molecule-induced transcriptomic perturbations are as accurate as *in vitro* experimental replicates.

**peaBrain imputed molecule profiles significantly outperform chemoinformatic/structure-based approaches in predicting drug approval.**

Having established the predictive capacity of the small molecule peaBrain models (on par with *in vitro* experimental replicates), we were interested in leveraging the peaBrain framework for drug repositioning. To this end, we imputed the expression profile for all clinically-relevant small molecules in the ChEMBL database(Gaulton et al. 2011) across all 5 cell lines (i.e. all molecules assigned at least to one indication). We divided all clinically-relevant molecules in two sets: molecules whose first approval occurred before the year 2000 (inclusive; n = 728 molecules), and all molecules that gained first approval after the year 2000 (n = 344 molecules). For indications relevant to each cell line (e.g. “breast neoplasms” for MC7 or “non-small cell lung carcinoma” for A549; **Table B,** *on next page*), we identified the subset of molecules assigned to the corresponding indication as the positive class (background is all other clinically-relevant molecules). We then trained a random forest classifier on the pre-2000 molecule set using the imputed peaBrain molecule profiles. We sought to assess how well the imputed peaBrain expression profiles can identify clinically-relevant molecules for a given indication, from all other molecules. To establish a baseline for comparison, we also constructed an equivalent classifier using only structural information (i.e. the molecule fingerprint used to impute expression). We assessed performance using four different metrics (useful metrics for classification with class imbalance):

1. F1 score (harmonic mean of precision and recall);
2. average precision (average of precisions achieved at each threshold weighted by the increase in recall from the previous threshold; corresponds roughly to the area under the precision-recall curve but is more conservative);
3. precision score (positive predictive value; the fraction of true positives among all molecules predicted as positive by the classifier); and
4. recall score (sensitivity; fraction of true positives over the number of all molecules relevant for the given indication).

All scores range from 0 to 1, with larger values indicating (elements of) better performance.

Across all five cell lines and the corresponding five indications, the imputed peaBrain expression profiles significantly outperformed chemoinformatic/structure-based approaches in predicting molecule clinical relevance for any given indication (**Table B**), with 64-86% increase in F1-scores, 22-63% increase in precision, 75-94% increase in recall, and 13-19% increase in average precision. peaBrain models that use molecule expression profiles from all cell lines have markedly increased performance compared to peaBrain models that use expression profiles from a single cell line, which in turn outperform structure-based approaches. We note that this trend for peaBrain performance is consistent even with more restrictive definitions for the positive class (i.e. when limited to molecules are that are exclusively labelled as clinically-relevant for the corresponding cell line indication and after removing molecules generally indicated for “neoplasms”).

| **Melanoma + Neoplasms (A375)** | structure-based approach | peaBrain (A375) | **% increase** | peaBrain (all) | **% increase** |
| --- | --- | --- | --- | --- | --- |
| F1-score | 0.12 | 0.14 | **20.37** | 0.27 | **78.97** |
| Average Precision | 0.37 | 0.42 | **12.05** | 0.42 | **10.84** |
| Precision Score | 0.40 | 0.70 | **54.55** | 0.52 | **25.35** |
| Recall Score | 0.07 | 0.08 | **15.38** | 0.18 | **90.91** |
| **Non-small cell lung carcinoma + Neoplasms (A549)** | structure-based approach | peaBrain (A549) | **% increase** | peaBrain (all) | **% increase** |
| F1-score | 0.14 | 0.18 | **26.93** | 0.29 | **71.79** |
| Average Precision | 0.36 | 0.40 | **9.99** | 0.43 | **15.52** |
| Precision Score | 0.44 | 0.64 | **38.02** | 0.55 | **22.49** |
| Recall Score | 0.08 | 0.10 | **25.00** | 0.20 | **83.33** |
| **Colorectal neoplasms + Neoplasms (HT29)** | structure-based approach | peaBrain (HT29) | **% increase** | peaBrain (all) | **% increase** |
| F1-score | 0.17 | 0.18 | **6.90** | 0.33 | **65.19** |
| Average Precision | 0.39 | 0.39 | -0.99 | 0.46 | **15.82** |
| Precision Score | 0.50 | 0.45 | **9.52** | 0.63 | **22.22** |
| Recall Score | 0.10 | 0.11 | **10.53** | 0.22 | **75.86** |
| **Breast neoplasms + Neoplasms (MCF7)** | structure-based approach | peaBrain (MCF7) | **% increase** | peaBrain (all) | **% increase** |
| F1-score | 0.14 | 0.33 | **83.74** | 0.35 | **89.18** |
| Average Precision | 0.38 | 0.37 | -3.26 | 0.46 | **18.87** |
| Precision Score | 0.32 | 0.50 | **43.90** | 0.62 | **64.06** |
| Recall Score | 0.09 | 0.25 | **96.77** | 0.25 | **96.77** |
| **Prostatic neoplasms + Neoplasms (PC3)** | structure-based approach | peaBrain (PC3) | **% increase** | peaBrain (all) | **% increase** |
| F1-score | 0.18 | 0.25 | **30.73** | 0.30 | **51.15** |
| Average Precision | 0.43 | 0.39 | -9.22 | 0.44 | **2.22** |
| Precision Score | 0.50 | 0.61 | **19.61** | 0.56 | **11.11** |
| Recall Score | 0.11 | 0.15 | **33.33** | 0.21 | **62.07** |

**Table B.** Tabulated classifier summary statistics on the post-2000 clinically-relevant molecule set (after training on the pre-2000 molecule set) for all five indications. For each indication, we constructed two peaBrain models: one using the expression profiles for the indication-relevant cell line and one using the expression profiles for all cell lines (denoted by “all”). The % increase columns highlight the increase in performance of the peaBrain classifiers compared to the baseline structure-based approach. The “all” peaBrain models significantly outperform all other approaches; the single cell line peaBrain models also have markedly increased performance compared to the baseline structure-based approach. We observe that this performance is consistent even with more stricter definitions for class indications (after removing molecules generally indicated for the MeSH term “neoplasms”).

**shRNA extension of the peaBrain DNA-reporter model captures ~85% of the variance in perturbed gene expression, allowing us to identify 292 new transcription factor-target interactions.**

Using a nearly identical model and assessment procedure, we can also predict the transcriptomic consequences of shRNA. Instead of a molecular fingerprint, we one-hot encoded the shRNA oligo-sequence as input, alongside the promoter sequence:

promoter + shRNA 🡪 expression

To highlight the utility of the shRNA model, we sought to predict the transcriptomic consequences of 330,617 unique shRNA oligo-sequences (a subset of which targeted 19,992 human genes) to enable inference of the human regulatory network; the extensive number of conditions provides (multiple) snapshots of the transcriptome with nearly every gene perturbed. We used a meta-inference approach (aggregation of multiple inference approaches) for network reconstruction, limited to genes directly measured on the L1000 platform (978 genes; see **Methods**).

Using the HTRIdb(Bovolenta et al. 2012) (an open-access database for experimentally verified human transcriptional regulation interactions) as the gold standard, we assessed our network inference for all 5 cell lines using a 5-fold Monte Carlo validation scheme with 10% of edges as test set (in every repeat). We noted the regulatory networks for each of the 5 cell lines captured experimentally-verified interactions at a precision ranging from 66-77%, with AUROC ranging from 87-93%, AUPRC from 39-52%, recall from 26-37% and F1-scores from 14-45% (**Fig B**). For comparison, the “Dialogue on Reverse Engineering Assessment and Methods” (DREAM) project constructed high-confidence networks for *Eschierica coli* and *Saccharomyces cerevisiae* (simpler organisms) at a precision of ~50%(Marbach et al. 2012). The inferred networks, for all 5 cells, have been made available (see **URLs in S4 Text**).

**Figure B** (*on next page*). Plots depicting the performance of network inference using the large-scale shRNA-perturbed expression matrix, across all 5 cell lines. Boxplot for each metric was generated using a 5-fold Monte Carlo validation scheme (with 10% of edges randomly selected as the test set). *Abbreviations:* AUROC, area under the receiver operating curve; AUPRC, area under the precision-recall curve.


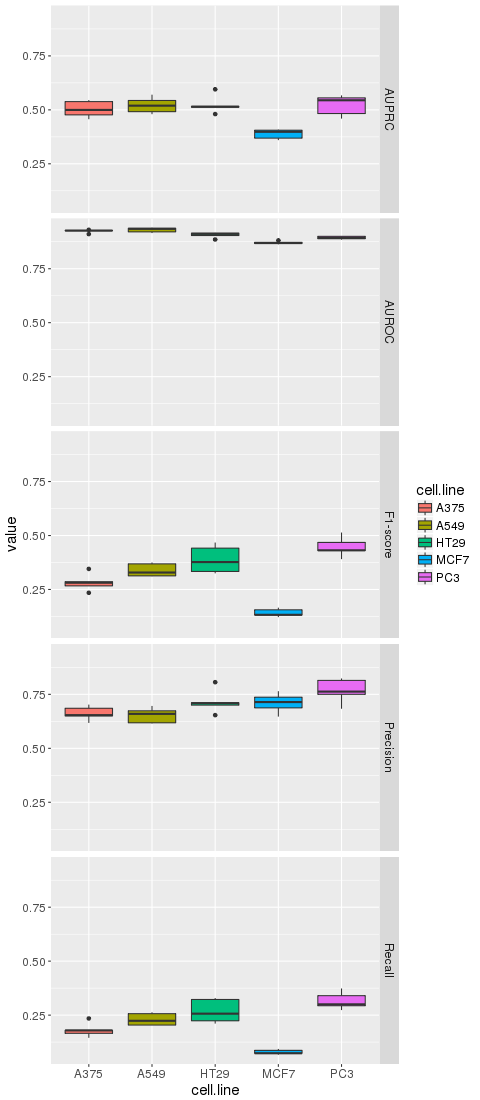


We subsequently identified all edges inferred as interactions – but absent from the experimentally-verified HTRIdb curation – as new/candidate regulatory interactions (n = 212 total). Using ChIP-seq data from ENCODE(Consortium 2007), we verified that 83% of have experiment support; that is, the predicted transcription factor binds within 50kb of the transcription start site of the corresponding target. Of all 212 interactions, 40% bind within 2kb of the transcription start site. All new inferred interactions have been made publicly available (see **URLs in S4 Text**).

**REFERENCES**

Bovolenta LA, Acencio ML, Lemke N. 2012. HTRIdb: an open-access database for experimentally verified human transcriptional regulation interactions. *BMC genomics* **13**: 405.

Consortium EP. 2007. Identification and analysis of functional elements in 1% of the human genome by the ENCODE pilot project. *nature* **447**: 799.

Gaulton A, Bellis LJ, Bento AP, Chambers J, Davies M, Hersey A, Light Y, McGlinchey S, Michalovich D, Al-Lazikani B. 2011. ChEMBL: a large-scale bioactivity database for drug discovery. *Nucleic acids research* **40**: D1100-D1107.

Marbach D, Costello JC, Küffner R, Vega NM, Prill RJ, Camacho DM, Allison KR, Aderhold A, Bonneau R, Chen Y. 2012. Wisdom of crowds for robust gene network inference. *Nature methods* **9**: 796.

Rogers D, Hahn M. 2010. Extended-connectivity fingerprints. *Journal of chemical information and modeling* **50**: 742-754.

Subramanian A, Narayan R, Corsello SM, Peck DD, Natoli TE, Lu X, Gould J, Davis JF, Tubelli AA, Asiedu JK. 2017. A next generation connectivity map: L1000 platform and the first 1,000,000 profiles. *Cell* **171**: 1437-1452. e1417.
