# Supplementary material for: A general framework for predicting the transcriptomic consequences of non-coding variation and small molecules
Source: PLoS Comput Biol. 2022 Apr 14;18(4):e1010028. doi: 10.1371/journal.pcbi.1010028 (PMC9041867; doi:10.1371/journal.pcbi.1010028)
Supplement: S4 Text — (DOCX) [file pcbi.1010028.s015.docx]

**S4 Text: List of URLs referenced in main text**

**Primary URL:**

peaBrain code and tutorials, <https://zenodo.org/record/6400074>

**Other URLs:**

BiT-STARR-seq, <https://www.biorxiv.org/content/early/2017/09/27/193136.figures-only>

CADD v1.3, <http://cadd.gs.washington.edu/download>

COSMIC v82, <https://cancer.sanger.ac.uk/cosmic/download>

DeepSEA, http://deepsea.princeton.edu/job/analysis/create/

Eigen v1.1, <http://www.columbia.edu/~ii2135/download.html>

Global Lipids Genetics Consortium, <http://csg.sph.umich.edu/abecasis/public/lipids2013/>

GTEx, <https://www.gtexportal.org/>

GTRD v17.04, <http://gtrd.biouml.org/>

HiDRA GEO, <https://www.ncbi.nlm.nih.gov/geo/query/acc.cgi?acc=GSE104001>

LDSC Epigenetic Annotations, <https://data.broadinstitute.org/alkesgroup/>

MPRA Supplemental Table, <https://www.ncbi.nlm.nih.gov/pmc/articles/PMC4957403/bin/NIHMS787218-supplement-7.xlsx>

phyloP, <http://hgdownload.soe.ucsc.edu/goldenPath/hg19/phyloP100way/>

Roadmap Annotations, <http://egg2.wustl.edu/roadmap/web_portal/>

RegulomeDB, <http://www.regulomedb.org/downloads>

Transcription factor binding sites (allele-specificity), <https://www.biorxiv.org/content/early/2018/02/01/253427.figures-only>
