# Supplementary material for: A general framework for predicting the transcriptomic consequences of non-coding variation and small molecules
Source: PLoS Comput Biol. 2022 Apr 14;18(4):e1010028. doi: 10.1371/journal.pcbi.1010028 (PMC9041867; doi:10.1371/journal.pcbi.1010028)
Supplement: Attachment — Submitted filename: Reply to Editor and Reviewers.docx [file pcbi.1010028.s016.docx]

Dear Dr Gamazon, Dear Dr Ioshikhes, Dear Dr Zhou and Other Reviewers,

Happy New Year! We hope our reply finds you and your loved ones healthy and well!

Thank you for considering and reviewing our manuscript "A general framework for predicting the transcriptomic consequences of non-coding variation and small molecules". We have incorporated all the revisions and have updated the manuscript accordingly. The comments and requested analyses were insightful and have been helpful in further refining our results – and we believe the manuscript is much better for it.

Please find our point-by-point reply below; the original review and comments are in blue and our reply is in black.

Changes and additions to the manuscript are highlighted in yellow.

***********************

Reviewer's Responses to Questions

**Comments to the Authors:**

Reviewer #1: The authors tackle predicting transcriptional regulation at the tissue level to clarify the consequences of genetic variation. They develop a convolutional neural network framework, peaBrain, which they apply for a series of predictive tasks, such as identifying variants with a regulatory function or predicting allele-specific transcription factor binding sites. They show that peaBrain explains most of the variance in mean transcript abundance when DNA and tissue-specific annotations are employed. They also compare its performance with that of elastic net and deep-learning based frameworks, such as DeepSEA or MPRA.

The demonstrated predictive performance of peaBrian is convincing. The analysis steps are also extensively detailed and the choices involved are thoroughly justified. On the practical utility of the method: it seems that peaBrain can help reveal general insights, such as on the relevance of given tissues for a complex trait of interest, but it is less clear that it can provide finer resolution to the functional mechanisms involved. Nonetheless, the work tackles an important and timely topic, and the manuscript is well written.

I have the following additional comments/questions:

1. Current state of research: The field of deep learning for transcriptome prediction is rapidly evolving, but the references cited in the manuscript all date back to 2018 or earlier. The authors should revise the literature review presented in the introduction so it reflects the current state-of-the-art. They should also confirm and indicate why their proposal is still competitive in light of the most recent work.

Our references initially often attempted to include two citations for each class of methods: (a) one citation referencing the earliest work and (b) one citation for the best-performing general (i.e., not tissue-specific) method. However, we whole-heartedly agree with the Reviewer and have expanded our reference list and comparisons to include:

Avsec, Z., Agarwal, V., Visentin, D., Ledsam, J.R., Grabska-Barwinska, A., Taylor, K.R., Assael, Y., Jumper, J., Kohli, P. and Kelley, D.R., **2021.** Effective gene expression prediction from sequence by integrating long-range interactions.

Agarwal, V. and Shendure, J., **2020.** Predicting mRNA abundance directly from genomic sequence using deep convolutional neural networks.

Kelley, D.R., **2020.** Cross-species regulatory sequence activity prediction. PLoS computational biology, 16(7), p.e1008050.

Ji, Y., Zhou, Z., Liu, H. and Davuluri, R.V., **2021.** DNABERT: pre-trained Bidirectional Encoder Representations from Transformers model for DNA-language in genome. Bioinformatics, 37(15), pp.2112-2120.

Lu, Q., Powles, R.L., Abdallah, S., Ou, D., Wang, Q., Hu, Y., Lu, Y., Liu, W., Li, B., Mukherjee, S. and Crane, P.K., **2017**. Systematic tissue-specific functional annotation of the human genome highlights immune-related DNA elements for late-onset Alzheimer’s disease.

Ghandi, M., Mohammad-Noori, M., Ghareghani, N., Lee, D., Garraway, L. and Beer, M.A., **2016.** gkmSVM: an R package for gapped-kmer SVM. Bioinformatics, 32(14), pp.2205-2207.

All of these additions are referenced appropriately in the introduction as well. At the suggestions of other reviewers, we’ve also expanded our analysis to include comparisons with Xpecto (2020), GenoSkyline (2017, suggested by Reviewer 3, Dr Zhou; lines 355-359), one new kmer-based variant scoring method (in addition to the one already included; lines 298-312) and one new position-weighted matrices approaches (in addition to the two already included; lines 298-312). Our new comparisons highlight why peaBrain is still competitive in light of the most recent work. We also included some discussion and brief comparison with DeepMind’s Enformer (2021; lines 706-713).

2. Interpretability: To what extent finer level insights can be obtained from the output of peaBrain? It is well understood that machine learning approaches can have excellent predictive capacity yet this advantage often comes at the cost of interpretability due to their overparametrised nature. In particular, the comparison of peaBrain with other methods for the different tasks A-H lacks of insights as of to which feature(s) of the framework leads to its improved predictive performance. In this context, it seems difficult to completely rule out overfitting, although the cross-validation procedures used in peaBrain as well as the corroborative evidence from other studies and biological knowledge alleviate this concern. Comments on this would be helpful. Similarly, it would be informative to discuss why the competing methods perform poorly or fail to reach a performance comparable to that of peaBrain for the different tasks considered (e.g., lines 143-145). Such insights would provide an additional motivation for deploying the method in practice.

We added three new analyses focused solely on interpretability. In particular, we sought to: (a) determine which feature(s) of the framework leads to peaBrain’s improved predictive performance; (b) design new and extensive comparative experiments to highlight how and why competing methods perform poorly or fail to reach a performance comparable to that of peaBrain for the different tasks considered; and (c) using the parameters of peaBrain (i.e., the learnt weights of the neural network layers) in various unrelated tasks to further refine what insights, aside from expression prediction, can be derived from the model. By using the model weights in unrelated tasks, we also provide further validation for the generalizability of the model (and by corollary, the lack of overfitting). Together, these experiments highlight what finer level insights can be obtained from peaBrain, increase the interpretability of the model architecture/design despite its (over)parameterized nature, and demonstrate peaBrain’s utility in practice. For convenience, we’ve copied the analyses below; two new figures (4 and 5) are in the main text.

(lines 260 – 282) **DNA sequence, annotated with experimentally-derived TFBS, from core promoter sequences are insufficient to predict mean abundance with high accuracy – epigenetic/histone markers contain the bulk of the information and are not readily accessible from the DNA sequence alone.** We were interested in determining the contribution of epigenetic/histone makers, alongside more general genomic annotations (such as coding sequences), in predicting the mean abundance of genes. In particular, we wanted to explore whether the DNA sequence alone was sufficient to predict expression. We noted that increasing the number of convolutional layers or the number of filters did not improve model performance (**Figure 4**). Explicitly incorporating TFBS into the model (i.e. annotating the DNA only and explicitly with TFBS) only improved performance slightly (cv-r2 = 23%), and was still considerably worse than the full class B model with epigenetic/histone marker annotations (cv-r2 = 46%; **Figure 4**). (Class-A DNA-only models had an average cv-r2 of 16% for skeletal muscle; class-C models annotated with tissue-specific information had an average cv-r2 of 57%.) The TFBS were collected from the Gene Transcription Regulation Database (GTRD) v17.4 with data on 476 human transcription factors and included peak calling with four different software (MACS, SISSRs, GEM, and PICS). In addition to including the processed peak calls, we also incorporated clusters (i.e. peaks merged for the same transcription factor but under different experimental conditions) and meta-clusters (i.e. non-redundant peaks synthesized from all four methods). This absence of improvement suggests that peaBrain model already recognizes many of the TFBS; identified by the convolutional filters inherent to the model architecture. These results indicate that experimentally-derived epigenetic and genomic annotations add information to that contained in the DNA sequence alone. As described above, this is broadly consistent with the observation that other convolutional neural networks models like DeepSEA are better at predicting TFBS (median AUC = 0.958) than at predicting histone modifications (median AUC = 0.856) [8, 33].

(lines 284 – 296) **peaBrain score out-performs existing measures in predicting allele-specific transcription factor binding.** As with tasks A and B, we compared the performance of the non-tissue-specific peaBrain score to predictions by CADD and EIGEN in predicting allele-specific binding, after accounting for allele frequency and evolutionary conservation. We assessed performance of the three non-coding metrics across 6675 sites in core promoter regions after filtering for duplicate sites [32]; 1896 of which exhibited allele-specific binding at an unadjusted binomial p < 0.05 (see **Methods**). We noted that only peaBrain impact score was significantly predictive of allele-specific binding sites (coefficient = 35.38 [12.00, 58.67]; p = 0.003; see Table 1); relaxing the binomial p-value threshold (i.e. increasing the number of sites considered as allele-specific) brings the other non-coding metrics to significance. peaBrain’s discriminative ability to identify allele-specific binding sites is consistent with our earlier observation that explicitly adding TFBS annotations did not improve the model. Notably, peaBrain’s ability indicates that average expression of all genes in a single tissue and the reference genome is sufficient to learn both TFBS and allele-specific binding.

(lines 298 – 312) To further investigate peaBrain’s ability to identify allele-specific binding sites, we compared peaBrain impact scores to predictions by methods specifically designed to predict TFBS, including two neural-network methods (DeepBind[29] and DeepSEA[8]), two kmer-based variant scoring methods (gkmSVM[30] and GERV[31]), and three position-weighted matrices (PWM)-related methods[32]. These methods depend on modelling TF ChIP-seq data in various ways and may have multiple models for the same TF. After confirming the predictive ability of these methods to identify allele-specific binding sites, we noted that peaBrain scores positively correlated only with GERV measures, a kmer-based variant scoring algorithm (Figure 5). Unlike the other methods, peaBrain (and GERV) do not assume the existence of canonical motifs and learn TFBS by modelling sequences (or kmers) directly (i.e., not simply by modelling the absence or presence of a ChIP-seq peak). In contrast, for both DeepBind and DeepSEA, we noted positive correlation with at least one PWM-method. These methods generally assume the existence of canonical TF binding sites and predictions are based on the extent of perturbation of those motifs. While this comparison is limited to variants for which data was available, the peaBrain results suggest that explicitly characterizing TF motifs is not necessary to understand the consequences of sequence variation on TF binding and transcriptional dysregulation.

The final analysis of using the parameters of peaBrain (i.e., weights of neural network layers) in various unrelated tasks was already part of the manuscript (in **Supplementary Note 1**), but we expanded the work to further highlight the performance of the weights. By using the model weights, we demonstrate inherent biological meaning to the parameters, further supporting both the architectural design of the model and the absence of overfitting. Both the manuscript (lines 361-372) and **Supplementary Note 1** have been updated to reflect this expanded work.

3. Tradeoff between computational efficiency and parameter choices: peaBrain relies heavily on cross validation and the authors repeatedly mention tradeoffs between computational tractability and accuracy. A runtime/memory profiling with respect to the sample size and for the different tasks A-H would be informative. This is particularly important as many recent studies involve samples sizes that are order of magnitude larger compared to GTEx. Also the choice of the 4 kbps core promoter window seems largely dictated by computational constraints, yet the performance is sensitive to this choice (lines 423-442). To alleviate this, it seems sensible to use tissue-specific windows; currently the optimisation of the window length was based on a single tissue (skeletal muscle). Would this improve predictive performance?

We have expanded the text to include more detailed runtime/memory profile; this builds on our previous discussion of computational tractability:

(lines 447-455) Complete analysis of a 1Mbps interval (including 5 different train/test splits) for a single gene in a single tissue and 94 individuals, if run sequentially on a CPU, required 15 days with 14 GB of memory. Limited to the genes with significant non-zero heritability in LCLs (n = 816), on 600 cores, the complete analysis took approximately a month. (Stage 1 peaBrain models only required several hours.) Prior to training, for each individual, we re-constructed the 1Mbps input sequence from the variants called from whole genome sequence (WGS) data (see Methods). Exploring the peaBrain architecture, fine-tuning the model parameters, and deploying the models was conducted on NVidia’s P100 GPUs (see Methods for details); the bulk of the training, however, was run on CPUs.

(NB: This added text is part of a larger revision that was added to address Reviewer 3’s comments so the entire section spans lines 436-455, but only lines 447-455 are relevant to this point.)

To explain the choice of the 4kb window, we reference below a Figure in Supplementary Note 2 (NB: this is only part of the figure for the sake of brevity):

(a) This is the structure of the Stage 1 peaBrain model (i.e., given a promoter and tissue, predict the average expression of the gene) – it only requires 4kbs promoter sequence as input. This takes only several hours to train and test.

(b) This is the structure of the Stage 2 peaBrain model (i.e., given two genotypes [or two different individuals], it predicts the difference in expression). This requires as input a 1Mbps (including the 4kbps core promoter sequence). This takes several weeks to train and test comprehensively as noted above.

By having this modular structure, we are able to initialize/transfer the learning done from the smaller (more computationally inexpensive models) to the larger more costly ones. The reason of 4kbs was that it performed sufficiently well for the promoter prediction task (stage 1) and we sought to leverage this training with the functional eQTL screening model (stage 2). The reviewer astutely gets at this idea of transferability in a point below and we actually leveraged it to our advantage. The manuscript, as well as the supplementary note, has been updated to reflect this.

4. Data: GTEx constitute a rich resource for assessing the performance of peaBrain in a tissue-centric fashion. However it also requires careful considerations, in particular: (1) GTEx samples were collected post mortem which is prone to batch effects. Is there a way such effects can be diagnosed from the peaBrain output? The authors indicate that the RPKM values were adjusted for list of study parameters, which should alleviate the issue, yet this is at the expense of true biological variation being removed as well; (2) how sensitive is peaBrain to population stratification? Here the first genotypes PCs were adjusted for, but it would nevertheless be interesting to evaluate the robustness of peaBrain to different degrees of stratification (or at least provide some discussion around how CNNs may be impacted by this issue); (3) the sample sizes for certain (less accessible) tissues can be rather small, e.g., for brain n < 250. In this case, the choice of k = 10 folds for the cross-validation scheme employed in peaBrain appears very large. For the elastic net model, the authors use k = 3 instead. What justifies these choices?

(1) and (2): To avoid overfitting (which could also be a consequence of batch effects in the dataset and/or overparameterization of the model), we employed several strategies inherent to the architecture of the model, including l2 and l1 regularization and extensive dropout. L2/l1 regularization introduces a new term to the regular loss function that is optimized by the neural network. It ensures that, independent of the gradient of the loss function, we are making our weights a little bit smaller each time an update is performed. A dropout layer, which we placed before the penultimate layer of the peaBrain architecture, is a another regularizer that randomly zeros input values (i.e. randomly dropping units and their connections), limiting co-adaptation and improving model generalizability (Hinton et al. 2012, Srivastava et al. 2014). This means that peaBrain is inherently insensitive to population stratification, but also by corollary, it means that peaBrain will reduce batch effects as the regularization drives the batch/noise signal down. In other words, by ensuring the model is robust to batch effects/noise, we unfortunately eliminated its ability to “label” them. (If there are batch effects, the model with extensive regularization and dropout readily adjusts for this – and drives the noise/batch effects to zero. This is on top of the adjustment for the genotype PCs and other batch effects, as suggested by the curators of the GTEx dataset.) We’ve added the following text to discussion and methods to highlight these features. We would also like to note that we are currently exploring diluting these batch-defeating measures to be able to analyze their effects as part of further work!

(3) We have re-run our models with n = 10 for elastic net; the text and figures have been updated! Our initial motivation for n = 3-10 for elastic net was that the variance was so small for some of the models that there was little difference between the runs. However, we increased n to 10 for all versions to be consistent with all other assessments.

5. Transferability & deployment: The analyses presented in the manuscript suggest that the insights obtained with peaBrain may be relevant to a large panel of applications. The range of scenarios tested, including the additional experiments of Supplemental Notes 2 and 3 (page 16), suggests that the method is both transferable and extensible. The code is provided to the reader, however peaBrain involves a large number of parameter choices, whose specification likely require expert knowledge or, at the very least, extensive experience. For the method to be adopted by the community, it thus seems important to provide thorough guidance on the method’s settings under the form of a tutorial or a software package with dedicated help pages. Also, the framework relies on external annotations (tissue-specific or not) — for general annotations, could they be provided to the user or easily linked when the method is called by user?

We have developed tutorials at: https://www.well.ox.ac.uk/~moustafa/peaBrain.shtml .

Screenshots of selected tutorials, provided in the form of python notebooks, are copied below. We hope to add several more tutorials and documentation before publication.

Re external annotations: Due to some of the data-sharing agreements that govern our access to the data, we are unable to provide the data directly (or methods that will access our institutional archive of the data), as that would be construed as hosting the data and would be in violation. In lieu of this, we now provide the processed data (i.e., how the model would take them as input data) at, as well as the function to read the data in:

All the data required to generate the results (in the form of the processed data) is available on the website.

6. Sharing information: Would peaBrain-like CNN architectures benefit from leveraging information across genes and/or tissues (rather than being applied separately for each gene/tissue) to exploit co-regulation patterns? Would such set-up be hampered by computational constraints?

peaBrain would definitely benefit extensively from leveraging information across genes and tissues – rather than being applied separately for each gene/tissue. We discussed part of this above – leveraging training from quicker-to-train models to speed up the training of computationally-expensive versions. We also apply this in multiple ways as noted by the author:

We leverage the sharing of information to predicting the impact of small molecules and shRNA (translating coregulation across small molecule effects to predict expression molecules of similar structure). While this analysis was already part of the manuscript (**Supplementary Note 3**), we expanded our focus on the “sharing of information”. We copy the first paragraph below (but the full analysis spans 11 double-spaced pages, with figures):

**Small molecule extension of the peaBrain DNA-reporter assay captures ~84% of the variance in the average molecule perturbed gene expression profiles of L1000 landmark transcripts.** To predict the transcriptomic perturbations of small molecules, we extended the peaBrain DNA reporter assay to incorporate molecular fingerprints (boolean arrays/bitmaps [i.e. a list of 1s and 0s] that are characteristic of the molecule structure), in addition to the DNA core promoter sequence as input (which directly accounted for copy number and genotypic variation, see Online Methods). *This task demonstrates the utility of the modular structure of peaBrain and the ‘shareability’ of information that is a consequence of its architecture/design.* As highlighted in Table 1, the small molecule extension can be described as:

promoter + molecular fingerprint 🡪 expression

where the fingerprint can be thought of as the “genotype” of the molecule. Given a core promoter sequence and a molecular fingerprint, the small molecule peaBrain model will predict the expression of the corresponding gene. We limited our analysis to molecular fingerprints – encoded as a standard bit vectors – generated using Morgan’s algorithm(Rogers and Hahn 2010) from the molecular-input line-entry system (SMILES) specification for each molecule. For each molecule profile (at 10uM dosage), we rank-normalized expression (averaging the replicates) and min-max scaled (per gene) prior to training (see Online Methods for details).

Aside from the experiments described above, we’ve also expanded the utility of the architecture in **Supplementary Note 2** and how different parts of peaBrain can ‘learn’ from each other.

Reviewer #2: In this manuscript, Abdalla et al. described a tool (peaBrain) to predict transcriptomic consequences of non-coding variants and small molecules. I appreciate the endeavor for showing the general statistics about the comparison of their model with others and how the prediction is consistent with the general biological interpretation. However, I feel it lacks detailed examples about the exact non-coding variants (exact position, etc) and their effect on which diseases. For instance, how many predicted functional variants were validated by experiments in the literature. This is extremely important for attracting a wide spectrum of readers.

Some specific comments:

Figures need to be improved. Fonts are too small. The authors need to make figures more compact to save space.

We have revised all the figures to increase the font size and decrease their size (reduced the white space to make the more compact).

Is peaBrain freely available for academic users? Can the authors provide detailed instruction for how to use peaBrain?

peaBrain is freely available for academic users at: https://www.well.ox.ac.uk/~moustafa/peaBrain.shtml

We’ve provided detailed instructions on the webpage and step-by-step instructions in extensive readme files contained in the download, alongside all relevant code and sample data. As requested by Reviewer 1, we have added several new tutorials (screenshots above). The Methods section in the manuscript also now contains the same information but formatted within the constraints of PLOS Computational Biology requirements. The new addition to the Methods are highlighted in yellow.

Reviewer #3: Moustafa et al. proposed a comprehensive framework for predicting the transcriptomic consequences. By incorporating the non-linearity feature from CNN, the authors successfully showed an insightful picture for gene expression regulation. The framework will be potentially very useful in multiple fields as the authors pointed out. However, details for the performance comparisons should be clarified to fully convince the readers.

Major comments

1. Line 46-47. Did the authors miss some earlier works (e.g., PMID 32433972) that performed cell-type-specific gene-expression predictions based on sequences? The Xpresso seems to be comparable with the step one of the proposed framework.

Xpresso uses our code, architecture, and has identical set-up. In fact, they cite our pre-print. We didn’t compare because we would be comparing peaBrain to itself. We have added this to the introduction (lines 66-70). We have also copied a screenshot of google scholar below confirming this:

2. Line 86. A brief description of the “peaBrain variant” will be helpful. Did the dataset used to define the “peaBrain variant” overlap with the one used for eQTL analysis?

Line 86 (now line 96) previously stated “the estimated peaBrain variant effects”, which we meant as the effect sizes of eQTL variants estimated using peaBrain. We have re-worded this accordingly to make it clearer.

3. Line 142-143. (1) The regularized linear model is not quite comparable in this case.

Agreed. We only include the regularized linear model as a simple baseline model (i.e., that a linear model can not recapitulate the performance of the Stage 1 peaBrain models). We have revised the text (lines 151--153) to state: *“… experimental models that we generated using regularized linear models (as a simple baseline model) fitted with stochastic gradient descent exhibited poor performance.”* Supplementary Note 1 has been similarly edited as well.

4. The correlation between the “non-coding impact score” and the conservation score is interesting. If I understand correctly, the loci with high impact scores are not necessarily to be loci of common variants. Studies had been shown that the gene expression level of conserved genes tends to be less predictable by cis variants. The relationships among conservation, allele frequency, and expression predictability (or cis heritability) would be an interesting follow-up.

Agreed. We have added a new analysis to explore the performance of non-tissue-specific peaBrain scores to predict allele-specific binding site. (We also included this analysis at the suggestion of Reviewer 1.) The analysis is copied below for convenience:

(lines 284 – 296) **peaBrain score out-performs existing measures in predicting allele-specific transcription factor binding.** As with tasks A and B, we compared the performance of the non-tissue-specific peaBrain score to predictions by CADD and EIGEN in predicting allele-specific binding, after accounting for allele frequency and evolutionary conservation. We assessed performance of the three non-coding metrics across 6675 sites in core promoter regions after filtering for duplicate sites[32]; 1896 of which exhibited allele-specific binding at an unadjusted binomial p < 0.05 (see Online Methods). We noted that only peaBrain impact score was significantly predictive of allele-specific binding sites (coefficient = 35.38 [12.00, 58.67]; p = 0.003; see Table 1); relaxing the binomial p-value threshold (i.e. increasing the number of sites considered as allele-specific) brings the other non-coding metrics to significance. peaBrain’s discriminative ability to identify allele-specific binding sites is consistent with our earlier observation that explicitly adding TFBS annotations did not improve the model. Notably, peaBrain’s ability indicates that average expression of all genes in a single tissue and the reference genome is sufficient to learn both TFBS and allele-specific binding. To further investigate peaBrain’s ability to identify allele-specific binding sites, we compared peaBrain impact scores to predictions by methods specifically designed to predict TFBS, including two neural-network methods (DeepBind[29] and DeepSEA[8]), two kmer-based variant scoring methods (gkmSVM[30] and GERV[31]), and three position-weighted matrices (PWM)-related methods[32]. These methods depend on modelling TF ChIP-seq data in various ways and may have multiple models for the same TF. After confirming the predictive ability of these methods to identify allele-specific binding sites, we noted that peaBrain scores positively correlated only with GERV measures, a kmer-based variant scoring algorithm (Figure 5). Unlike the other methods, peaBrain (and GERV) do not assume the existence of canonical motifs and learn TFBS by modelling sequences (or kmers) directly (i.e. not simply by modelling the absence or presence of a ChIP-seq peak). In contrast, for both DeepBind and DeepSEA, we noted positive correlation with at least one PWM-method. These methods generally assume the existence of canonical TF binding sites and predictions are based on the extent of perturbation of those motifs. While this comparison is limited to variants for which data was available, the peaBrain results suggest that explicitly characterizing TF motifs is not necessary to understand the consequences of sequence variation on TF binding and transcriptional dysregulation.

While is only one analysis, we also hope to further explore the relationship between conservation, allele frequency, and expression predictability in future work.

5. Line 277. The sample size should be mentioned given the p-value.

Sample size was added (n = 5268). This is now line 231; we slightly re-organized some of the sections after revising to ensure readability and flow.

6. Line 330. The comparison between RTC and peaBrain is less comprehensive, compare with the quality in the other parts of the manuscript. Other tissue-prioritization methods need to be justified. e.g., PMID 27058395

We now include a comparison with GenoSkyline, as suggested by the reviewer. The full results are in **Supplementary Table 4.** Interestingly, both peaBrain and GenoSkyline identified liver, monocytes, adipose, and gastrointestinal tissue as the primary tissues for total cholesterol, in the same rank order (Supplementary Table 4) – further supporting the validity of both approaches. The analysis added to the main text is copied here for convenience; the comparison is noted in italics:

**Tissue-specific peaBrain scores can identify the functional tissues underlying GWAS signals from complex traits.** For tasks A-C, we have used the non-tissue-specific peaBrain score (average of score, per position, across all tissues) to facilitate comparison with the other tissue-agnostic impact metrics. However, we sought to investigate advantages of tissue-specific impact scores. In particular, we wanted to highlight how tissue-specific scores could allow us to identify functional tissues associated with GWAS signal from complex traits (task D). We hypothesized that the “true” functional gene(s) downstream of a GWAS locus (“hit”) would have, on average, higher peaBrain impact scores for the tissue in which the gene is likely to act, given that >50% of the variance in mean gene abundance can be explained by the promoter sequence. In other words, we hypothesized that genes associated with a given phenotype (e.g. total cholesterol) are also likely to be transcriptionally perturbed in the underlying functional tissue (e.g. liver), which we can detect with tissue-specific peaBrain scores. For brevity, we selected 4 quantitative traits[34] (total cholesterol, LDL, HDL, and triglycerides) for which the (primary) putatively causal tissue is well-established and included in the GTEx dataset. Using HESS[35], we calculated the local SNP-heritability from the relevant GWAS summary statistics, while accounting for linkage disequilibrium. For European populations, HESS partitions the genome into 1703 approximately-independent LD blocks (average length = 1.6Mb)[35]. For each block (or “locus”), we calculated the tissue-specific peaBrain impact score for each GTEx tissue; the locus peaBrain score is defined as the average of the tissue-specific peaBrain scores at all positions (with a score) within that locus. We subsequently performed a regression of the rank-transformed local SNP-heritabilities as a function of the rank-transformed peaBrain locus scores to minimize bias caused by outlying loci and assessed significance for the linear model coefficient (n = 45 tests for each GTEx tissue per phenotype; see Methods). As a baseline benchmark, we compared our results to tissue predictions made using the tissue trait concordance (RTC) score[36], which was adapted to calculate the probability that a GWAS-associated variant and an eQTL are co-localized and weighted by the extent of tissue sharing for the given eQTL to obtain tissue-causality profiles for each trait. Across all tested traits, we noted the peaBrain framework was better at identifying putatively causal tissues than simply using the RTC-/eQTL-based method (Supplemental Tables 3 and 4). For LDL, using the peaBrain framework, the top five tissues (ranked by nominal p-value) were: EBV-transformed lymphocytes, visceral adipose, fibroblasts, liver, and terminal ileum (small intestine); all were significant after Bonferroni adjustment with p-values tabulated in Supplemental Table 3. In contrast, with the RTC-based method, the top five tissues were: sun-exposed skin (from lower leg), pancreas, fibroblasts, tibial nerve, and cerebellar hemisphere (brain). This was consistent across all tested traits (e.g. for HDL, liver ranked 3rd using the peaBrain framework and 32nd using the RTC-based method; **Supplemental Table 4**). The superior peaBrain performance suggests inherent limitations to eQTL-based methods that are sidestepped by the Stage 1 peaBrain framework, which depends only on the average expression of all genes in a single tissue and the reference genome. Notably, peaBrain is independent from the number of eQTLs identified per tissue and the number of genome-wide significant hits for a given trait, which are both limitations for eQTL-GWAS co-localization methods (such as the RTC-based framework). *Further confirming this hypothesis, we observe that GenoSkyline-Plus[37], a tool that uses expanded set of epigenomic and transcriptomic annotations to produce high-resolution, single tissue annotations (rather than eQTLs alone), identifies that all 4 lipid traits shared a similar enrichment pattern in liver, adipose, and monocyte tissue (Supplementary Tables 3 and 4 ), consistent with the causal relationship among these traits observed with the peaBrain analysis. Interestingly, both peaBrain and GenoSkyline identified liver, monocytes, adipose, and gastrointestinal tissue as the primary tissues for total cholesterol, in the same rank order (Supplementary Table 4) – further supporting the validity of this approach.*

We would also like to draw attention to expanded supporting analyses in Supplementary Note 2, extending these methods to other tissues and traits.

7. Line 460. The best performing cis window size for expression abundance across genes and variance across individuals seems to be inconsistent (4k v.s. 1m).

We’ve clarified that the *functional eQTL screen model* (i.e., the Stage 2 peaBrain model that uses 1MBps as input to predict) is actually an extension of the Stage 1 *promoter sequence reporter* peaBrain model (that uses 4kb as input). The reason why we expand to 1M (from 4kb) is because differences/variation between two individuals is relatively rare in the 4kb surrounding the representative TSS of any given gene*. We need to expand the model to capture this variation, but we use the same architecture. It is sufficient to use the 4kb sequence for the promoter models because there is sufficient differences between promoters of any two given genes. We have expanded the text and Supplementary Note 2 to clarify this modular extension. A portion of the figure, illustrating the overlap in model architecture, is copied below:

* We quantified the heritability of 4kbs vs 1Mbps regions (i.e., their ability to predict transcriptomic differences between individuals) and added the following analysis to the text as supporting evidence:

(lines 436 – 455) Consistent with evidence from eQTL studies[38], we noted the 4kbps core promoter used in Stage 1 did not capture enough cis-heritability as estimated by constrained GCTA[39] and thus was not sufficiently informative for this predictive task. In LCLs, for example, using the 4kbps core promoter, genes with significant non-zero heritability (p < 0.01; n = 1066) had a median heritability of 0.136. We selected a 1Mbps input length, centred on the annotated TSS (0.5Mbps upstream and 0.5Mbps downstream), as a compromise between computational tractability (extending the sequence entails more computational expense) and biological relevance (the potential to capture additional narrow-sense heritability with extended intervals). Using the 1Mbps input sequence, genes with significant non-zero heritability (p < 0.01; n = 816) had a median heritability of 0.270; nearly twice the heritability captured with the core promoter 4kbps sequence. Importantly, our symmetric 1Mbps window likely contained >95% of cis-eQTLs; in the GTEx dataset, the 95th percentile for absolute distance of cis-eQTLs from their target transcript TSS was 441,698bps[24]. Complete analysis of a 1Mbps interval (including 5 different train/test splits) for a single gene in a single tissue and 94 individuals, if run sequentially on a CPU, required 15 days with 14 GB of memory. Limited to the genes with significant non-zero heritability in LCLs (n = 816), on 600 cores, the complete analysis took approximately a month. (Stage 1 peaBrain models only required several hours.) Prior to training, for each individual, we re-constructed the 1Mbps input sequence from the variants called from whole genome sequence (WGS) data (see Methods). Exploring the peaBrain architecture, fine-tuning the model parameters, and deploying the models was conducted on NVidia’s P100 GPUs (see Methods for details); the bulk of the training, however, was run on CPUs.

**Supplementary Note 2** further details the overlap of the architecture, including how the model architecture is both transferable and extensible, and the modular design can enable users to benefit from leveraging information across genes and/or tissues to exploit coregulation patterns, within reasonable computational constraints.

8. Line 470. To my knowledge, the poor performance using elastic net is not consistent with the PrediXcan paper (Figure 3). More details should be provided. A r2 (elastic net) v.s. r2 (peaBrain) scatter plot would be very helpful to visualize to improvment. Ideally, training the prediction model in GTEx LCLs and estimating the performance in GEUVADIS LCLs.

PrediXcan and elastic net models perform well for large sample sizes. Prof Frank Harrel (professor of biostatistics at Vanderbilt) notes that performance of elastic net (and other penalized estimation models) will heavily shrink coefficient estimates to the mean, i.e., the estimates will be conservative, and thus performance will suffer when the sample size is small. However, the intercept in the model is not shrunk, and therefore, our peaBrain model performs better for tissues with smaller sample sizes. (The other added benefit of peaBrain, unlike elastic net is that peaBrain suggests likely functional variant from the subset of eQTLs in LD, which elastic net cannot do.) We have added the above details to the manuscript.

We’ve also expanded our analysis, including more detailed comparison with elastic net and deepSea. We performed the training on GTEx LCLs and tested on both European and Nigerian GEUVADIS LCLs separately. The analysis is copied below; please note that the first paragraph overlaps slightly with the older analysis but is provided for convenience/readability:

(lines 488-498) Having established the predictive ability of peaBrain, we were interested in whether we can use the best-performing peaBrain models to measure the impact of single variants, compared to DeepSEA log fold change (logFC) estimates and experimental log skew estimates from MPRA and BiT-STARR-seq (Task F). For all “captured” genes (n = 113), we selected all variants identified as significant eQTLs in the GTEx v6p univariate eQTL analysis (n = 16,019 variants; see Methods) and replicated the analysis with the Geuvadis dataset [44] (n = 17,279 variants for the EU population and n = 1601 variants for the YRI [Yoruba from Ibadan, Nigeria] population). Unlike GTEx v7, eQTLs from GTEx v6p were derived from genotyping arrays (Illumina OMNI 5M or 2.5M) and thus did not include WGS used to train the peaBrain model. The Geuvdais dataset (both EU and YRI populations) were eQTLs derived from WGS from a set of non-overlapping subjects. For each eQTL (including indels), we created pairs of artificial sequences that only differed at the corresponding snp/indel position and predicted the difference in expression between the alternate and reference alleles from the difference between the two artificial sequences. (We used only a single model of the those several trained during cross-validation for simplicity, but incorporating results from additional models improves results; see **Methods**.)

(lines 503-535) peaBrain predictions significantly and positively correlated with the univariate eQTL coefficients from the GTEx analysis (Spearman’s rho = 0.09; p = 3.02 x10^-32^; Supplementary Figure 2), from the EU-Geuvadis analysis (rho = 0.10; p = 9.60 x10^-38^; **Supplementary Figure 3**), and from the YRI-Geuvadis analysis (rho = 0.18; p = 8.64 x10^-13;^ **Supplementary Figure 4**). Results were consistent across all datasets when we relaxed our heritability to include genes with GCTA p < 0.05: Spearman’s rho equal to 0.08 (p = 3.02 x10^-25^), 0.07 (p = 3.65 x10^-25^), and 0.18 (p = 1.68 x10^-13)^ for the GTEx, EU-Geuvadis, and YRI-Geuvdais univariately-significant eQTLs, respectively. Alongside this positive correlation, we observed that many variants with large coefficients across all three datasets had small peaBrain predictions (Supplementary Figures 2-4). This shrinkage in peaBrain estimates is consistent with the appreciable LD between eQTL-associated variants at any given locus, such that only a subset of significantly-associated variants are actually functional. Whilst univariate linear models are not capable of distinguishing between functional versus “hitchhiker” variants, the joint modelling of the input sequence in Stage 2 peaBrain allows a more direct assessment of the function of each variant. In comparison, we noted that the MPRA log skew estimates did not correlate with the univariate eQTL coefficients in the GTEx (rho = 0.014; p = 0.60), the EU-Geuvadis (rho = -0.018; p = 0.58), or the YRI- Geuvadis (rho = 0.011; p = 0.82) eQTL analyses. The BiT-STARR-seq log skew estimates also did not correlate with the univariate eQTL coefficients in the GTEx (rho = -0.031; p = 0.50), the EU-Geuvadis (rho = 0.035; p = 0.44), or the YRI-Geuvadis (rho = -0.112; p = 0.32) eQTL analyses. Both the MPRA and BiT-STARR-seq experimental assays were run in lymphoblastoid cell lines. Similarly, for GM12878 (LCL) annotations, we noted that the maximum log fold changes from DeepSEA did not correlate with the magnitude of the eQTL coefficients in the GTEx analysis (rho = -0.001; p = 0.92), the EU-Geuvadis analysis (rho = -0.010; p = 0.18), and the YRI-Geuvadis (rho = 0.016; p = 0.52). The DeepSEA results suggest that simply predicting chromatin effects and TFBS is not sufficient for predicting the transcriptomic consequences of sequence variation for this set of selected genes. This is consistent with experimental evidence suggesting that most genetic variants in DNase footprinted (and other annotated) regulatory regions are silent [45], and the fact that histone modifications (e.g. methylation) alone are frequently insufficient to transcriptionally perturb promoters [46]. However, it is important to note that DeepSEA was trained on the reference genome (i.e., not exposed to genotype variation), while Stage 2 peaBrain was trained to predict differences in expression as a function of differences in sequence between any pair of individuals. Finally, and most importantly, we did not have any variant-level filters for any of the methods (e.g., using a p-value threshold for the experimental assays or any significance cut-off for the peaBrain estimates); thus, our comparison was not biased towards any method and assessed the utility of the method estimate across the range of variant effects.

9. A discussion for the very recent work from the DeepMind group will be welcome.

We have expanded our discussion to include the recent work from the DeepMind group, which has also been copied here for convenience:

(lines 710 – 717) Our initial explorations, using peaBrain, have demonstrated that regulatory activity up to 1Mbp away from TSS can predict differences between individuals for a subset of genes. The next stage of this work is design more efficient and computationally inexpensive models. Transformers, which are at the heart of DeepMind’s enformer, provide one option: modelling relationships between genomic positions regardless of their respective position. However, as we have shown, the position of the variants also encodes valuable observation. As such, transformers provide only part of the solution; new methods must both model the influence of enhancers and distant regulatory regions as well as retain their positional information (which is necessary to identify person-to-person differences).

Minor comments

1. Line 55. Is Expecto computationally too expensive to have a larger cis window size?

That is what the authors of Expecto state; Expecto includes only single nucleotide substitutions within 1kb to the representative TSS of a gene. We have copied a screenshot from the accompanying webpage below:

2. Line 153. Type ‘OOS’.

Corrected. It now states: *OSS R^2^ = out-of-sample r^2^* . (This line is now in a separate location for the Figure captions, at the end of the manuscript.)

3. Line 386. ‘F1-score’ should be defined for people do not familiar with the ML terminologies.

Added a definition for F1-score: *the harmonic mean of precision and recall,* *a measure of model performance and accuracy that is robust to class imbalance* (now lines 400-402).

4. Line 416. The non-linear feature may also result from the activation function of CNN.

Agreed. We added this to the text as well. The new line now states: “By jointly modelling the input “difference” sequence in a non-linear manner *(as a result of the activation function of the CNN)*, …” (This is line 428 after the revisions.)

**Have the authors made all data and (if applicable) computational code underlying the findings in their manuscript fully available?**

The [PLOS Data policy](https://urldefense.proofpoint.com/v2/url?u=https-3A__journals.plos.org_ploscompbiol_s_materials-2Dand-2Dsoftware-2Dsharing&d=DwMGaQ&c=WO-RGvefibhHBZq3fL85hQ&r=9zD_KcxLuwhXiYuUMz0OJ5IHc4q9q7xyB61iclykVtOypua0Pp4iKFphrsf3SX7x&m=xDa8FcPGv9TPjGFEHgE8II9Iq727vzXkac6t4Ce2YP7NQuCbzr-UhesviCD1hCMl&s=yVhrSbp8sGgL3F3xcU8eY6uE-_P6YgOtzP_0whJpQsk&e=) requires authors to make all data and code underlying the findings described in their manuscript fully available without restriction, with rare exception (please refer to the Data Availability Statement in the manuscript PDF file). The data and code should be provided as part of the manuscript or its supporting information, or deposited to a public repository. For example, in addition to summary statistics, the data points behind means, medians and variance measures should be available. If there are restrictions on publicly sharing data or code —e.g. participant privacy or use of data from a third party—those must be specified.

Reviewer #1: Yes

Reviewer #2: None

Reviewer #3: None

PLOS authors have the option to publish the peer review history of their article ([what does this mean?](https://urldefense.proofpoint.com/v2/url?u=https-3A__journals.plos.org_ploscompbiol_s_editorial-2Dand-2Dpeer-2Dreview-2Dprocess-23loc-2Dpeer-2Dreview-2Dhistory&d=DwMGaQ&c=WO-RGvefibhHBZq3fL85hQ&r=9zD_KcxLuwhXiYuUMz0OJ5IHc4q9q7xyB61iclykVtOypua0Pp4iKFphrsf3SX7x&m=xDa8FcPGv9TPjGFEHgE8II9Iq727vzXkac6t4Ce2YP7NQuCbzr-UhesviCD1hCMl&s=UlSiMiVLZp4ECbwERZPjI1bQ7wR95jyPtpSfI-aACjI&e=)). If published, this will include your full peer review and any attached files.

If you choose “no”, your identity will remain anonymous but your review may still be made public.

**Do you want your identity to be public for this peer review?** For information about this choice, including consent withdrawal, please see our [Privacy Policy](https://urldefense.proofpoint.com/v2/url?u=https-3A__www.plos.org_privacy-2Dpolicy&d=DwMGaQ&c=WO-RGvefibhHBZq3fL85hQ&r=9zD_KcxLuwhXiYuUMz0OJ5IHc4q9q7xyB61iclykVtOypua0Pp4iKFphrsf3SX7x&m=xDa8FcPGv9TPjGFEHgE8II9Iq727vzXkac6t4Ce2YP7NQuCbzr-UhesviCD1hCMl&s=ghattlc9X5HMsvhoBEs3_jYvW4dt-g2TCiUVkRC7NkM&e=).

Reviewer #1: No

Reviewer #2: No

Reviewer #3: **Yes:** Dan Zhou

Figure Files:

While revising your submission, please upload your figure files to the Preflight Analysis and Conversion Engine (PACE) digital diagnostic tool, [https://pacev2.apexcovantage.com](https://urldefense.proofpoint.com/v2/url?u=https-3A__pacev2.apexcovantage.com_&d=DwMGaQ&c=WO-RGvefibhHBZq3fL85hQ&r=9zD_KcxLuwhXiYuUMz0OJ5IHc4q9q7xyB61iclykVtOypua0Pp4iKFphrsf3SX7x&m=xDa8FcPGv9TPjGFEHgE8II9Iq727vzXkac6t4Ce2YP7NQuCbzr-UhesviCD1hCMl&s=ELCRkhHJdw-T1-a918k-Z3LeKot6YsmRkGlEQIojpRg&e=). PACE helps ensure that figures meet PLOS requirements. To use PACE, you must first register as a user. Then, login and navigate to the UPLOAD tab, where you will find detailed instructions on how to use the tool. If you encounter any issues or have any questions when using PACE, please email us at [figures@plos.org](mailto:figures@plos.org).

Data Requirements:

Please note that, as a condition of publication, PLOS' data policy requires that you make available all data used to draw the conclusions outlined in your manuscript. Data must be deposited in an appropriate repository, included within the body of the manuscript, or uploaded as supporting information. This includes all numerical values that were used to generate graphs, histograms etc.. For an example in PLOS Biology see here:

http://www.plosbiology.org/article/info%3Adoi%2F10.1371%2Fjournal.pbio.1001908#s5.

Reproducibility:

To enhance the reproducibility of your results, we recommend that you deposit your laboratory protocols in protocols.io, where a protocol can be assigned its own identifier (DOI) such that it can be cited independently in the future. Additionally, PLOS ONE offers an option to publish peer-reviewed clinical study protocols. Read more information on sharing protocols

at [https://plos.org/protocols?utm_medium=editorial-email&utm_source=authorletters&utm_campaign=protocols](https://urldefense.proofpoint.com/v2/url?u=https-3A__plos.org_protocols-3Futm-5Fmedium-3Deditorial-2Demail-26utm-5Fsource-3Dauthorletters-26utm-5Fcampaign-3Dprotocols&d=DwMGaQ&c=WO-RGvefibhHBZq3fL85hQ&r=9zD_KcxLuwhXiYuUMz0OJ5IHc4q9q7xyB61iclykVtOypua0Pp4iKFphrsf3SX7x&m=xDa8FcPGv9TPjGFEHgE8II9Iq727vzXkac6t4Ce2YP7NQuCbzr-UhesviCD1hCMl&s=1FNRH1nAmq1e0c-w-9xsdNnmZR3N7XY5toZS86YKJqY&e=)
