# Supplementary material for: A general framework for predicting the transcriptomic consequences of non-coding variation and small molecules
Source: PLoS Comput Biol. 2022 Apr 14;18(4):e1010028. doi: 10.1371/journal.pcbi.1010028 (PMC9041867; doi:10.1371/journal.pcbi.1010028)
Supplement: Attachment — Submitted filename: Reply.docx [file pcbi.1010028.s017.docx]

Dear Dr Gamazon, Dr Ioshikhes, and Reviewers,

I hope this reply finds you and your loved ones healthy and well!

Below, please find our point-by-point reply in black, with the original reviewer comment in blue. Changes to the manuscript are highlighted in yellow. As required, we also submitted a clean version of the manuscript with no highlights.

---

Reviewer #1: The authors have put a lot of effort into improving the manuscript, and all my questions have been addressed. No further comment.

Thank you!

Reviewer #2: The authors have addressed my comments. I recommend the acceptance of the revised manuscript.

Thank you!

Reviewer #3: The authors addressed most of my concerns. However, I didn’t find adequate information for the comparison between elastic net and peaBrain in gene expression prediction across individuals.

The authors still need to detail the claim that only 28 of the 816 cis-heritable genes can be captured using elastic net. It would be great if the authors can show a transcriptome-wide picture for the predictability comparison between peaBrain and elastic net when the sample size varies.

We added more details about the performance when the sample size varies (lines 489-497 and lines 488-505) and detail why only 28 of the 816 cis-heritable genes can be captured using elastic net (the very small number of samples [approximately 100 individuals]). In contrast, because peaBrain leverages differences between individuals, we have a combinatorial larger dataset (100 choose 2 = 4950 pairs of individuals). This gets at the core difference between the two approaches: exchanging model performance with computational costs. peaBrain performs incredibly well (even with very small datasets) but it is far more computationally expensive. Further, unlike linear models, peaBrain retains/encodes position of the variant in the sequence. Linear models (e.g., elastic net) are sufficiently reasonable with a large number of individuals and are very inexpensive to compute. For very large datasets, linear models may be more reasonable (depending on the desired accuracy and performance). However, with increasing availability of gpu clusters and decreasing computational costs, peaBrain may be more appropriate for small to medium-sized datasets. Lines 449-457 discuss the computational of the peaBrain approach, compared to a linear model. Lines 672-679 also explain mechanistically why peaBrain performs better on smaller sample sizes (i.e., retaining sequence information).
